# Co-overexpression of chitinase and β-1,3-glucanase significantly enhanced the resistance of Iranian wheat cultivars to Fusarium

**DOI:** 10.1186/s12896-024-00859-0

**Published:** 2024-05-24

**Authors:** Negin Mohammadizadeh-Heydari, Masoud Tohidfar, Bahram Maleki Zanjani, Motahhareh Mohsenpour, Rahele Ghanbari Moheb Seraj, Keyvan Esmaeilzadeh-Salestani

**Affiliations:** 1 Department of Agronomy and Plant Breading, Agriculture Faculty, Zanjan University, Zanjan, Iran; 2 Department of Cell and Molecular Biology, Faculty of Life Sciences and Biotechnology, Shahid Beheshti University, Tehran, Iran; 3 Department of Tissue Culture and Gene Transformation, Agricultural Biotechnology Research Institute of Iran (ABRII), Karaj, Iran; 4 Department of Horticultural Sciences, Faculty of Agriculture and Natural Resources, University of Mohaghegh Ardabili, Ardabil, Iran; 5 Chair of Crop Science and Plant Biology, Institute of Agricultural and Environmental Sciences, Estonian University of Life Sciences, Fr. R.Kreutzwaldi 1, 51014 Tartu, Estonia; 6 Institute of Technology, University of Tartu, Nooruse 1, E-50411 Tartu, Estonia

**Keywords:** Chitinase, β-1,3-glucanases, Biolistic method, Transgenic wheat

## Abstract

Fusarium head blight (FHB) is a devastating fungal disease affecting different cereals, particularly wheat, and poses a serious threat to global wheat production. Chitinases and β-glucanases are two important proteins involved in lysing fungal cell walls by targeting essential macromolecular components, including chitin and β-glucan micro fibrils. In our experiment, a transgenic wheat (*Triticum aestivum*) was generated by introducing chitinase and glucanase genes using Biolistic technique and Recombinant pBI121 plasmid (pBI-ChiGlu (-)). This plasmid contained chitinase and glucanase genes as well as nptII gene as a selectable marker. The expression of chitinase and glucanase was individually controlled by CaMV35S promoter and Nos terminator. Immature embryo explants from five Iranian cultivars (Arta, Moghan, Sisun, Gascogen and A-Line) were excised from seeds and cultured on callus induction medium to generate embryonic calluses. Embryogenic calluses with light cream color and brittle texture were selected and bombarded using gold nanoparticles coated with the recombinant pBI-ChiGlu plasmid. Bombarded calluses initially were transferred to selective callus induction medium, and later, they were transfferd to selective regeneration medium. The selective agent was kanamycin at a concentration of 25 mg/l in both media. Among five studied cultivars, A-Line showed the highest transformation percentage (4.8%), followed by the Sisun, Gascogen and Arta in descending order. PCR and Southern blot analysis confirmed the integration of genes into the genome of wheat cultivars. Furthermore, in an in-vitro assay, the growth of *Fusarium graminearum* was significantly inhibited by using 200 μg of leaf protein extract from transgenic plants. According to our results, the transgenic plants (T_1_) showed the resistance against Fusarium when were compared to the non-transgenic plants. All transgenic plants showed normal fertility and no abnormal response was observed in their growth and development.

**Supplementary Information:**

The online version contains supplementary material available at 10.1186/s12896-024-00859-0.

## Introduction

Wheat (*Triticum aestivum*) is one of the most important staple crops in the world, which is placed in the first rank because of its domestication and contribution for providing the primary food for human beings [1]. However, different pathogens and diseases negatively affect the wheat morphological and philological processes and reduce the quality and quantity of the yield [2]. The *Fusarium graminearum,* a predominant species of Fusarium head blight (FHB), is one of serious fungal diseases, which damages the yield and contaminates the grains with mycotoxins, threatening human and animal health [3–5]. Application of chemical fungicides is one of effective strategies to control the pathogenic fungi but it also targets beneficial organisms due to non-specificity, which in time cause environmental issues [6]. On the other hand, antifungal proteins, which prevent and suppress the growth and multiplication of pathogenic fungi, are produced by plants in response to fungal pathogens [7]. Chitinases and β-glucanases are two important proteins that lyse the cell walls of fungi by targeting substantial components such as chitin and β-glucan microfibrils [8].

Targeting the genes encoding hydrolytic enzymes are the best strategy for conferring genetic resistance against a wide range of plant fungal pathogens in transgenic crops [9, 10]. This approach provides many advantages including crops tolerance improvement against biotic and abiotic stresses and grain quality increment [11]. Biolistic as a high-performance method is commonly used to deliver foreign DNA and/or RNA directly into plant cells owing to its less physiological risk and no need to microbial intermediaries such as agrobacterium strains, requiring less additional DNA as well as being compatible with both monocotyledon and dicotyledonous plants [12].

Different studies have been done to increase the resistance of wheat cultivars to the Fusarium. The chitinase gene transferred to wheat resulted in Fusarium-resistant plants [13, 14]. In wheat, higher expression of glucanase led to increased resistance to Fusarium [15]. It was reported that the co-expression of chitinase and glucanase genes could increase the resistance to Fusarium in other plants [16, 17]. Since Iranian wheat cultivars are frequently infected by this destructive fungus, our aim was to investigate the effect of simultaneously transferred glucanase and chitinase genes through biolistic method against Fusarium disease.

## Materials and methods

### Design and construction of pBI-ChiGlu(-)^1^ vector

**Fig. 1:**
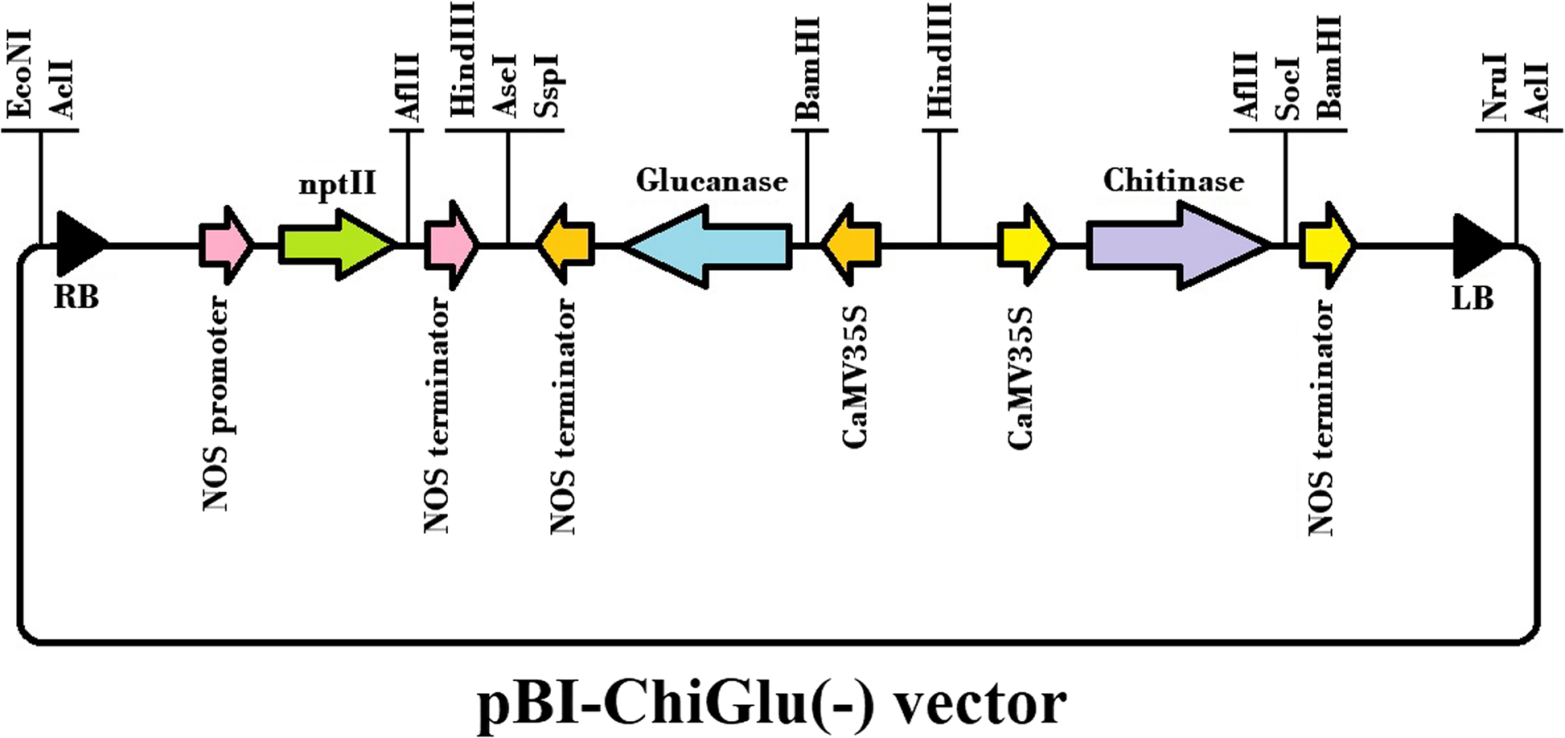
Schematic map of the T-DNA region of recombinant binary vector pBI-ChiGlu (-), carrying chitinase and glucanase genes driven by CaMV35S promoter (P35S) and nptII gene driven by Nos promoter. LB, left border; RB, right border; nptII, neomycin phosphotransferase

Sequences for chitinase (from beans) and glucanase (from barley) genes were obtained from the NCBI database and were used as templates for designing the primers using Vector NTI Software [18] (Fig. 1). Total RNA was extracted using RNeasy Plant Mini Kit (QIAGEN, Germany), followed by synthesis of the first-strand cDNA using QuantiTect Reverse Transcription Kit (QIAGEN, Germany) according to the manufacturer’s instructions. PCR amplification included an initial step of 95 °C for 5min, followed by 30 denaturation cycles at 95 °C for 1 min and primer annealing at 57 °C for 1 min and extension cycles at 72 °C for 1 min, and finally extension was done at 72 °C for 5 min. Then PCR products were visualized on a 1% agarose gel. The relevant bands were recovered from the gel and purified using QIAquick Gel Extraction Kit (QIAGEN, Germany). To create the sticky end, the genes were cloned into pCaMV plasmids and digested by HindIII and BamHI and visualized by 1% agarose gel. The bands with two sticky ends were again isolated from the gel and purified. Subsequently, glucanase and chitinase genes were cloned into pCaMV and pGEM vectors, respectively. Next, the pGEM-Chi plasmid was digested with *XbaI* and *SacI* enzymes, and the chitinase gene was recovered and ligated to the pBI121 plasmid. PCaMV-Glu plasmid was also digested by *HindIII* and the glucanase gene was recovered and cloned into pBI121-Chi plasmid. Finally, neomycin phosphotransferase as a selectable marker gene was ligated to the recombinant plasmid of pBI121-ChiGlu (-). The chitinase and glucanase genes were controlled by CaMV35S promoter and NOS terminator, and the neomycin phosphotransferase II (nptII) gene was controlled by NOS promoter and Terminator. The vector was constructed according to the Fig. 1.

### Plant materials and transfection procedure

The seeds of wheat cultivars including two spring genotypes (Arta and Mogan), two winter genotypes (Sisun and Gascogen) and A Line were obtained from the Seed and Plant Improvement Institute of Iran. The bombardments were performed using Biolistic PDS-1000/He Particle Delivery System and (900 or 1100 psi) rupture disc.

### Callus induction and genes insertion

The seeds were sown in pots with a mixture of peat and perlite (1/1) and then were kept under controlled conditions in the Phytotron at 26°C and photoperiod of 16 h light + 8 h of darkness. Twelve to fourteen days after anthesis immature seeds were collected and surface sterilized with 70% (v/v) ethanol for 5 min, followed by soaking in commercial sodium hypochlorite 2.5%(v/v) for 20 min. Then, seeds were rinsed and washed several times with sterile water. Immature embryos were excised microscopically in a sterile environment and placed with scutellum in upward direction on callus induction medium at 26°C in dark condition for 45 days. The medium for callus induction included the Murashige and Skoog (MS) salts and vitamins [19] supplemented with 2 mg/l 2,4-Dichlorophenoxyacetic acid (2,4-D), 4 mg/l Thiamine HCl, 200 mg/l Casein hydrolysate, 500 mg/l Glutamine, 30 g/l sucrose and 8 g/l agar at pH 5.8. Embryonic calluses were bombarded with 1μm gold particles, coated with 1μg/μl DNA of recombinant plasmid pBI-ChiGlu (-). After bombardment, embryonic calluses were kept overnight at 26°C in the dark condition. Then, the embryonic calluses were placed on selective callus induction media supplemented with 25 mg/l kanamycin and kept at 26°C in dark condition for a week.

### Regeneration of putative transgenic plantlets

Induced embryonic calluses were divided into smaller pieces and placed on a selective regeneration medium supplemented with 25 mg/l kanamycin at 26°C and photoperiod of 16 h light + 8 h of darkness for regeneration and production of shoots and roots. Regeneration medium included MS salts and vitamins supplemented with 2 mg/l BAP, 0.1 mg/l IAA, 30 g/l sucrose, and 7 g/l agar at pH 5.7. Plantlets with shoots and roots were transferred to a regeneration medium without kanamycin supplemented with 1.5 g/l activated charcoal, which were sub-cultured every two weeks. The percentage of shooting and rooting as well as transformation were measured. Regenerated plantlets were initially transplanted to small pots with a mixture of peat and perlite (1/1) and were kept under plastic bags for adaptation in the growth chamber. Then, they were transferred to big pots and kept in the normal greenhouse conditions at 26°C. Transgenic wheat lines (T_0_) were successfully self-pollinated, leading to the production of T_1_ seeds.

### Polymerase chain reaction (PCR) analysis

The PCR was performed for transgenic plants (T_0_) using specific primers listed in Table 1. For this purpose, fresh leaves were ground into the powder in presence of liquid nitrogen and were used for extraction of genomic DNA according to the Dellaporta method [20]. PCR reactions included 2.5 μl of 10X reaction buffer, 2 μl MgCl_2_ (25 mM), 2.5 μl dNTPs (0.2 mM), 10 ρM of each specific primer, 25 ng DNA, 1 U Taq DNA polymerase, and nuclease-free water up to 25 μl. The PCR was performed using CaMV35S forward and chitinase reverse primers as well as CaMV35S forward and gluconase reverse primers to confirm the presence of both chitinase and glucanase genes and their individual promoters, respectively. Thermal cycling conditions consisted of an initial denaturation at 94°C for 5 min, followed by 35 cycles of 94°C for 1min, 58–60°C for 30s, and 72°C 1 min as well as a final extension at 72°C for 5 min. In all reactions, water sample (PCR reaction without DNA template), non-transgenic wheat DNA and recombinant plasmid were used as contamination test, negative control, and positive control, respectively. The PCR products were evaluated by electrophoresis on a 1% agarose gel. Three replicates were considered for PCR analysis in each sequence.

**Table 1:** Primers used in PCR analysis of transgenic plants

**Sequence**	**Primers**	**PCR product length (bp)**	**Annealing temperature (**°C)
chitinase	F: GAGTGGTGTGGATGTTG R: GCCATAACCGACTCCAAGC	872	60
gluconase	F: CAGGTCCAAGGGCATCAACG R: CTCCGACACCACCACCTTC	629	60
nptII	F: GAACAAGATGGATTGCACGC R: GAAGAACTCGTCAAGAAGGC	786	58
CaMV35S	F: CCACGTCTTCAAAGCAAGTGG R: TCCTCTCCAAATGAAATGAACTTC	123	60
35S- chitinase	F: CCACGTCTTCAAAGCAAGTGG R: GCCATAACCGACTCCAAGCA	1096	60
35S- gluconase	F: CCACGTCTTCAAAGCAAGTGG R: TCTCCGACACCACCACCTTC	972	60

### Southern blot hybridization analysis

Non-radioactive Southern blot analysis was carried out according to the DIG Application Manual (Roche Diagnostics GmbH, Germany). Twenty μg of T_0_ wheat genomic DNA was digested using *Eco*RI at 37°C for an overnight. Digested DNA was run on a 0.8% agarose gel electrophoresis and blotted to a positively charged nylon membrane following the protocol (HAYBOND N + , Amersham, Little Chalfont, UK). PCR DIG probe synthesis and DIG detection kit were used to create the probe corresponding to a PCR product of gene and detection of the probes (Boehringer, Mannheim, Germany), respectively [21]. Three replicates were considered for southern blot hybridization analysis in each sequence.

### In vitro assay of Fusarium infection and macro- and microscopic analysis

Fresh leaves of T_1_ putative transgenic and non-transgenic wheat plants were ground into powder in presence of liquid nitrogen. The extraction of soluble proteins was performed using 10mM sodium acetate buffer (pH 7.0). Three replicates were considered for in vitro assay of each sample according to Tohidfar et al. [22] method. A single sclerotium of *F. graminearum* was placed on the center of petri dishes (9 cm diameter) comprising potato dextrose agar (PDA). For primary mycelia growth, the plates were incubated at room temperature. A mycelia plug (1 cm diameter) of fungus grown on the previous PDA medium was collected and placed on the center of a new PDA plate surrounded by marginal wells comprising 200 and 100 μg leaf protein extracts prepared in 10 mM sodium acetate buffer (pH 7.0) from the non-transformed wheat plant, 100 and 50 μl of extraction buffer, and 200 and 100 μg leaf protein extracts from the transgenic wheat plant. Plates were incubated at room temperature for 5 days, and macroscopic and microscopic approaches were used for analyzing the mycelia morphology and expansion inhibition zone.

### Gene expression analysis by Real-Time PCR

Two genes were selected for real-time PCR. Specific primers were designed using OLIGO Primer Analysis Software v.7.0 (National Bioscience Inc., Plymouth, USA). Real-time PCR with three technical and three biological replicates was done on a Rotor-Gene Q (QIAGEN, Germany) using SYBR® Green Fluorescent DNA Stain-low ROX (Jena Bioscience, Germany) according to the optimized program for each candidate gene. The reference gene was *actin* and the gene expression level was calculated using the Delta-Delta CT method [23] performed in the REST2009 software according to the comparative threshold cycle, and the graphs were made using the GraphPad Prism9(GraphPad Software, United States).

### Statistical analysis

Statistical analysis was performed using R (version 3.5.321). Data were analyzed using ANOVA, employing a completely randomized design where treatments were considered fixed effects and replicates as random effects. Mean values were subsequently compared utilizing the Duncan test function within the *agricolae* package, with significance set at a 5% probability level.

## Results

### Confirmation of cloned genes into plasmid vector

**Fig. 2:**
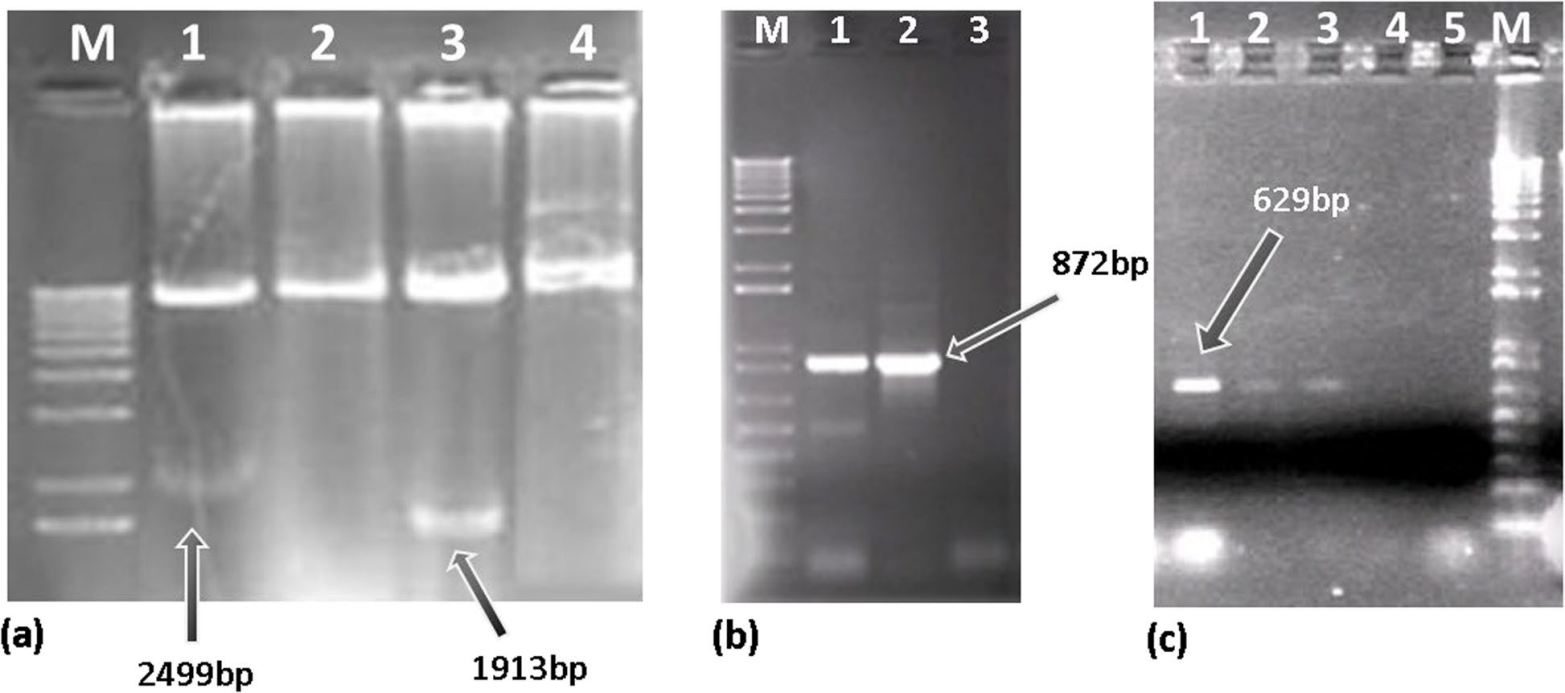
**a** Digestion of pBI-ChiGlu recombinant plasmids in order to verification of presence and insertion direction of chitinase and glucanase genes. M: size marker 1 Kb plus, Lanes 1 and 2: *Bam*HI digestion for pBI-ChiGlu(-) and pBI121-Chi respectively, Lanes 3 and 4: *Hind*III digestion for pBI-ChiGlu(-) and pBI121-Chi (–) respectively; **b** PCR analysis using specific primers for verification of chitinase gene presence. M: DNA size marker 1 Kb plus, Lane 1: pBI-ChiGlu(-), Lane 2: pBI121-Chi; Lane 3: Negative control (pBI-Glu); **c** PCR analysis using specific primers for verification of glucanase gene presence. Lane 1: pBI-Glu, Lanes 2, 3: pBI-ChiGlu; Lane 4: Negative control (pBI-Chi), Lane 5: Negative control (PCR reaction without DNA template), M: DNA size marker 1 Kb plus

Enzymatic digestion and PCR analysis were used to confirm genes cloning into the vector as well as to determine the direction of gene insertion. The *Bam*HI and *Hind*III enzymes were used to prove the presence of chitinase and glucanase genes in pBI-ChiGlu (-), respectively. Electrophoresis of PCR products on agarose gel confirmed the presence of bands with 872 and 629 bp lengths for chitinase and glucanase, respectively (Fig. 2).

### Transformation and regeneration of calluses

**Fig. 3:**
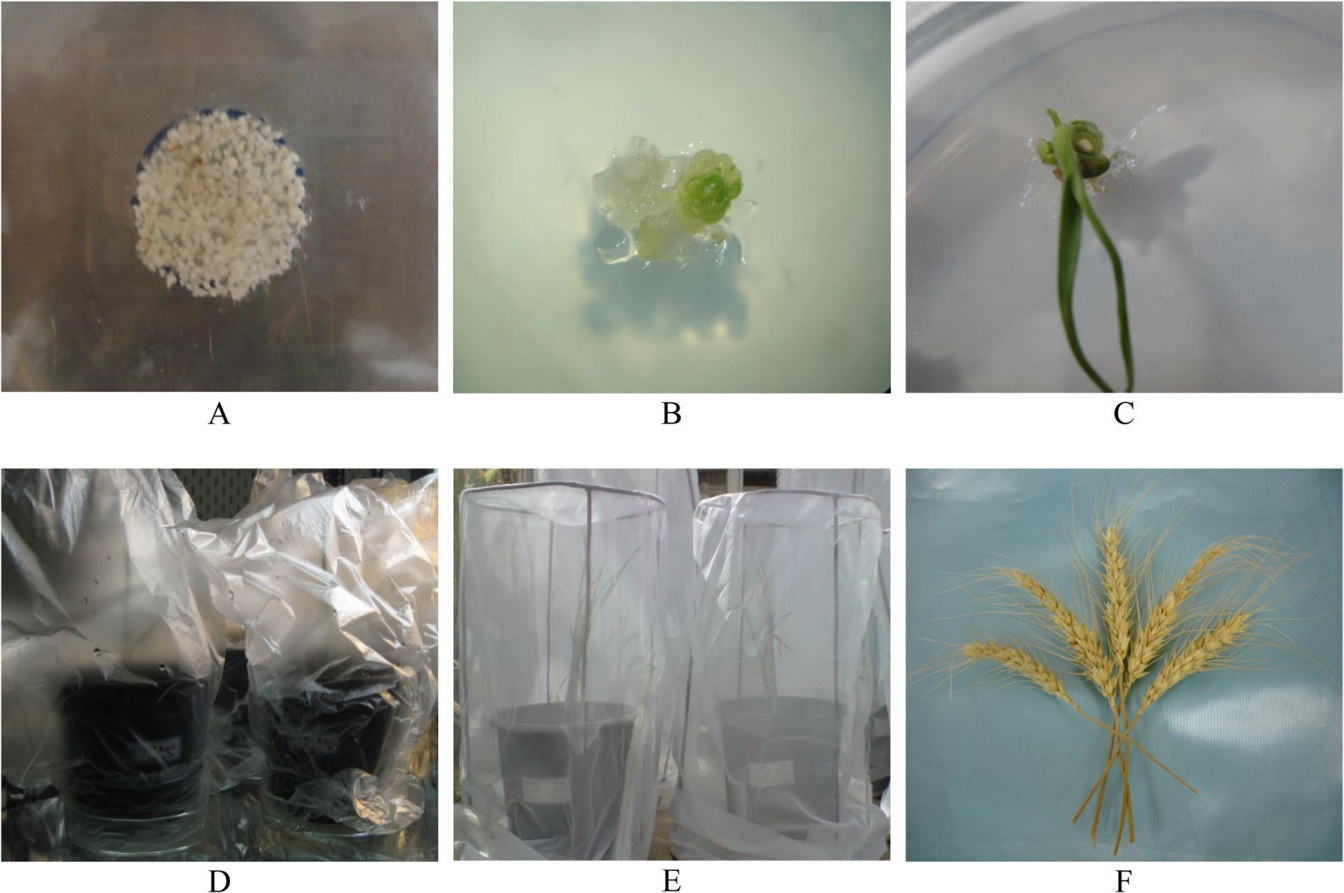
Regeneration of transgenic wheat. **A** Embryonic calluses after bombardment, **B** Somatic embryo on selection medium containing 25 mg/l of kanamycin, **C** Regeneration of somatic embryo, **D** Regenerated plants in pots under plastic bags, **E** Transgenic wheat plants transplanted to pots in greenhouse, **F** Transgenic plant seeds

In total, 2295 embryogenic calluses were bombarded, resulting in the production of 15 transgenic plantlets. Two plantlets belonged to cultivar whereas 13 plantlets were from cultivar. Regeneration percentages of Mogan and A Line were 0.3 and 4.8, respectively. The percentage of transformation and regeneration for three other cultivars was equal to zero (Table 2). The regeneration stages have been presented in Fig. 3.

**Table 2:** Percentage of shooting, rooting, transgenic plantlets and Transformation in different cultivars of Iranian wheat

Genotypes	Bombed calluses	Shooting percentage	Rooting percentage	Transgenic plantlets	*Transformation percentage
Sisun	135	0^d^	0^c^	0^c^	0^c^
Gascogen	90	0.8^c^	0^c^	0^c^	0^c^
Moghan	675	2.7^b^	3.1^b^	2^b^	0.3^b^
Arta	1125	1.4^bc^	2.8^b^	0^c^	0^c^
A Line	270	10.2^a^	11.7^a^	13^a^	4.8^a^

^*^Transformation percentage was obtained from the ratio of transgenic calli to the number of bombed calli

^a–c^Mean values with the same letter are not significantly different (*p* < 0.05)

### PCR analysis of transgenic plants

**Fig. 4:**
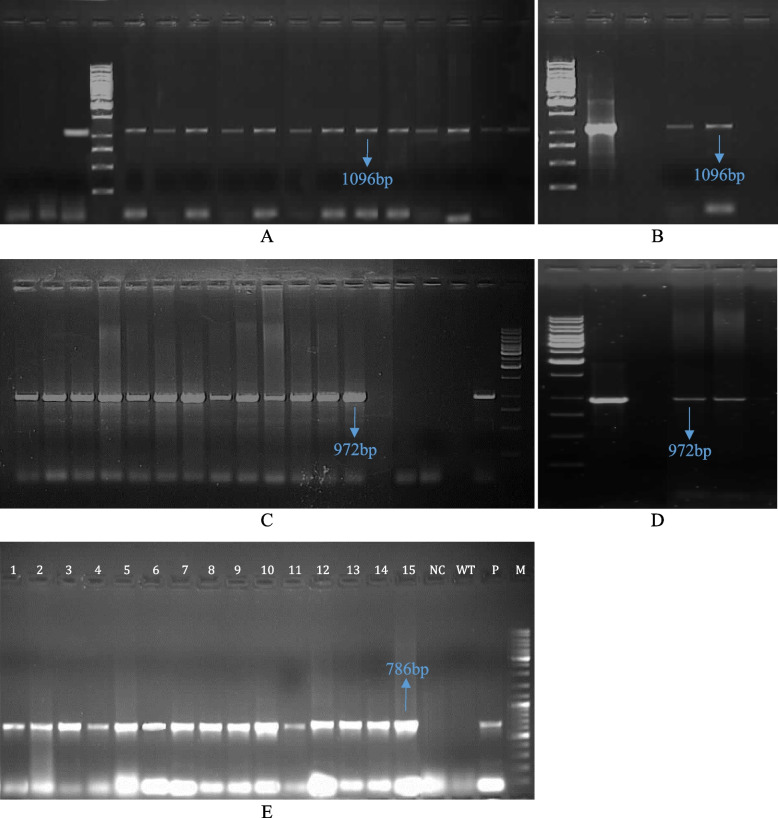
PCR analysis for T_0_ Putative transgenic wheat plants using different specific primers. **A** PCR analysis using CaMV35S forward and chitinase reverse primers for A Line cultivar (including 13 transgenic plantlets). **B** PCR analysis using CaMV35S forward and chitinase reverse primers for Moghan cultivar (including 2 transgenic plantlets). **C** PCR analysis using CaMV35S forward and glucanase reverse primers for A Line (including 13 transgenic plantlets). **D** PCR analysis using CaMV35S forward and glucanase reverse primers for Moghan (including 2 transgenic plantlets). **E** PCR analysis using *nptII* forward and reverse specific primers, Lanes 1–15 (including 13 A Line and 2 Moghan transgenic plantlets). M: DNA size marker (A, B, C, D: 1 Kb ladder fermentas; E, 1 Kb plus ladder); P: pBI-ChiGlu recombinant plasmid as positive control; WT: non-transformed control plant; NC: Negative control (PCR reaction without DNA template)

**Fig. 5:**
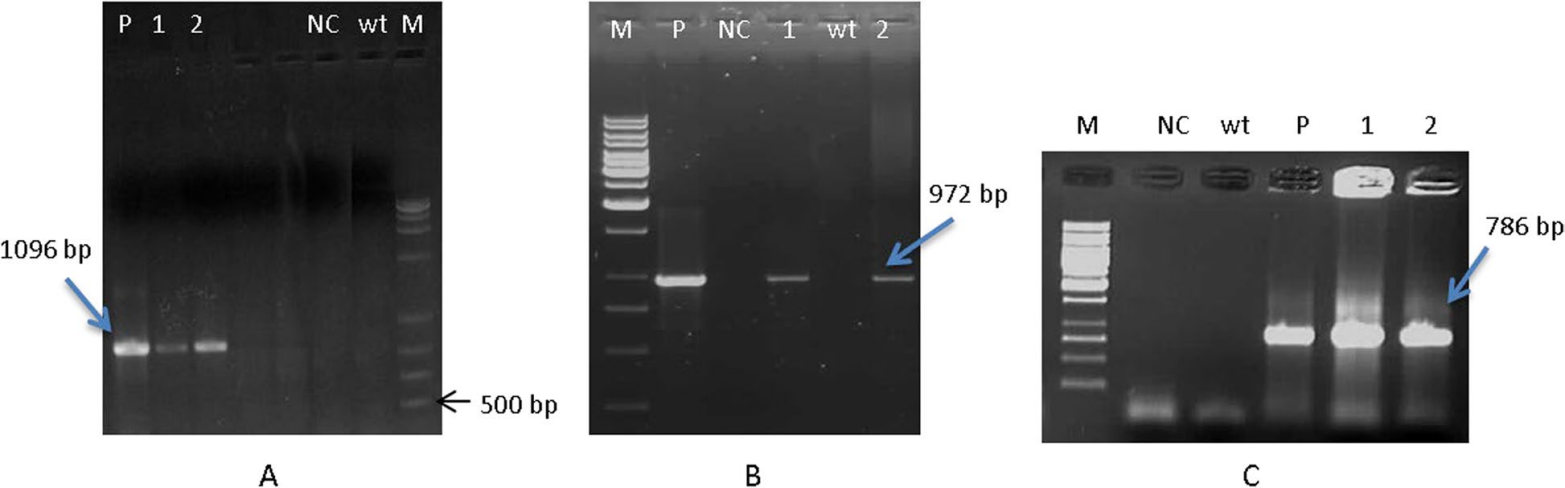
PCR analysis for T1 transgenic wheat plants of A Line using different specific primers. **A** PCR analysis using CaMV35S forward and chitinase reverse primers; **B** PCR analysis using CaMV35S forward and gluconase reverse primers; **C** PCR analysis using *nptII* forward and reverse specific primers. **A**, **B**, **C** Lanes 1 and 2: transgenic wheat plants; M: DNA size marker (1 Kb ladder fermentas); P: pBI-ChiGlu(-) recombinant plasmid as positive control; Wt: non-transformed control plant; NC: Negative control(PCR reaction without DNA template)

PCR analysis on T_0_ and T_1_ plants showed that all putative transgenic plants have received at least one copy of the genes (Figs. 4 and 5). Presence of fragments with 1096 bp, 972 bp, and 786 bp length for chitinase, glucanase, and nptII in T_0_ plants, respectively, were confirmed (Fig. 4) whereas no band was observed in non-transgenic plant and negative control. PCR analysis for T_1_ transgenic plants showed that the genes were inserted into the wheat genome (Fig. 5).

### Southern blot analysis of T_1_ transgenic plants

**Fig. 6:**
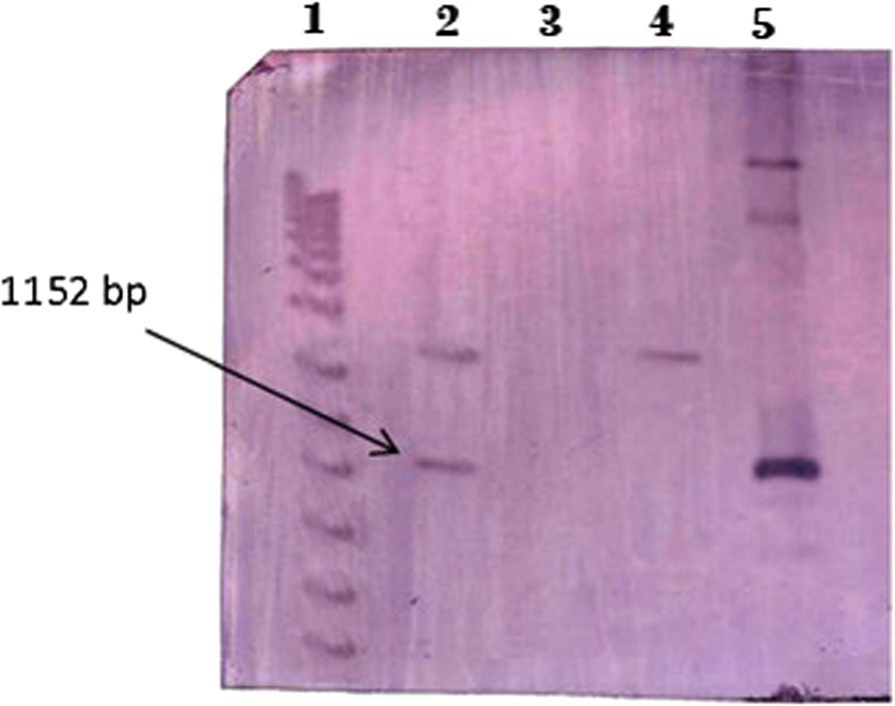
Southern blot analysis of T_1_ transgenic plants in A-Line transgenic wheat. Lane1: DNA Size marker 1 Kb; Lane 2: *Eco*RI digested DNA from transgenic event 1; Lane 3: non-transgenic plant; Lane 4: *Eco*RI digested DNA from event 2; Lane 5: *Eco*RI digested DNA from plasmid (pBI121-ChiGlu (-))

Southern blot analysis of T_1_ was employed to detect the presence of transgenes in the wheat genome. Since glucanase and chitinase were on the same vector and were transferred together, this analysis was performed for the chitinase gene in A-Line cultivar for two events. The results showed that event 1 and 2 had two and one copies, respectively. No bands were observed in non-transgenic plants. This analysis confirmed that events of the A-Line cultivar have at least one copy of the gene in its genome (Fig. 6).

### In vitro assay of Fusarium infection (macroscopic and microscopic analysis)

**Fig. 7:**
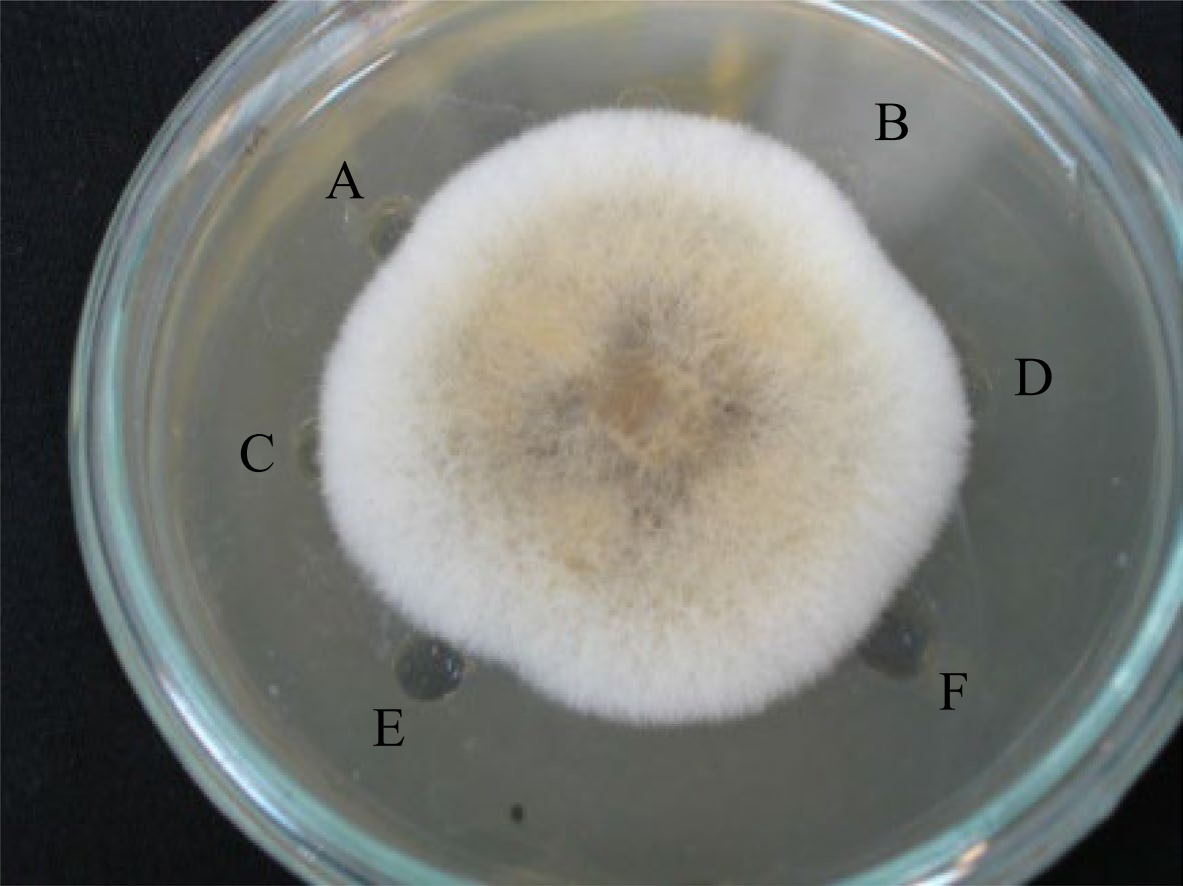
Macroscopic analysis of the inhibitory activity of leaf protein extract from T_1_ transgenic wheat, expressing chitinase and glucanase gene against Fusarium. Samples were loaded into each individual well at the periphery and fungal mycelia plug was placed in the center of the plate. Samples were as follows: (**A**) 200 μg and (**B**) 100 μg protein extract from leaf tissues of non-transgenic wheat, (**C**) 100 μl and (**D**) 50 μl of extraction buffer (10 mM sodium acetate buffer (pH 7.0)), (**E**) 200 μg and (**F**) 100 μg protein extract of transgenic wheat

**Fig. 8:**
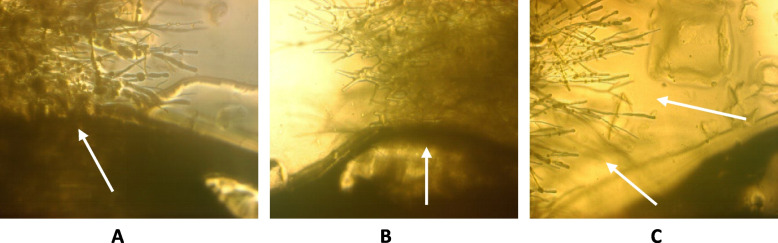
Microscopic analysis of the inhibitory activity of leaf protein extract from transgenic wheat on of Fusarium. **A** extraction buffer, **B** leaf extracts from the non-transgenic plant, **C** leaf protein extracts from the transgenic wheat, on mycelial growth of Fusarium, (**A**) and (**B**) showed that fungal mycelium has overgrown into the well on PDA and mycelia become visible normal, whereas in (**C**) mycelial growth has stopped and lysed before attainment the wells containing the transgenic leaf extract

The inhibitory properties of T_1_ transgenic plants are presented in Fig. 7. Application of 200 µg of the leaf protein extracts showed a significant positive impact on inhibition of fungal growth (macroscopic analysis). On the contrary, non-transgenic plant protein extracts and extraction buffer had no impact on the Fusarium growth (Fig. 7). Fungal activity was examined by microscopic analysis for further confirmation of the inhibitory effect of leaf protein extracts. The results showed that protein extract of A-Line cultivar inhibited the fungal growth through decomposition of hyphal tips, which stopped the growth of fungal mycelium towards the media, leading to weaker and thinner fungal hyphae. In the non-transgenic cultivar and extraction buffer media, the fungus showed persistent growth towards the media. It not only covered the surface of the media but also penetrated into it (Fig. 8).

### Real-time PCR analysis

**Fig. 9:**
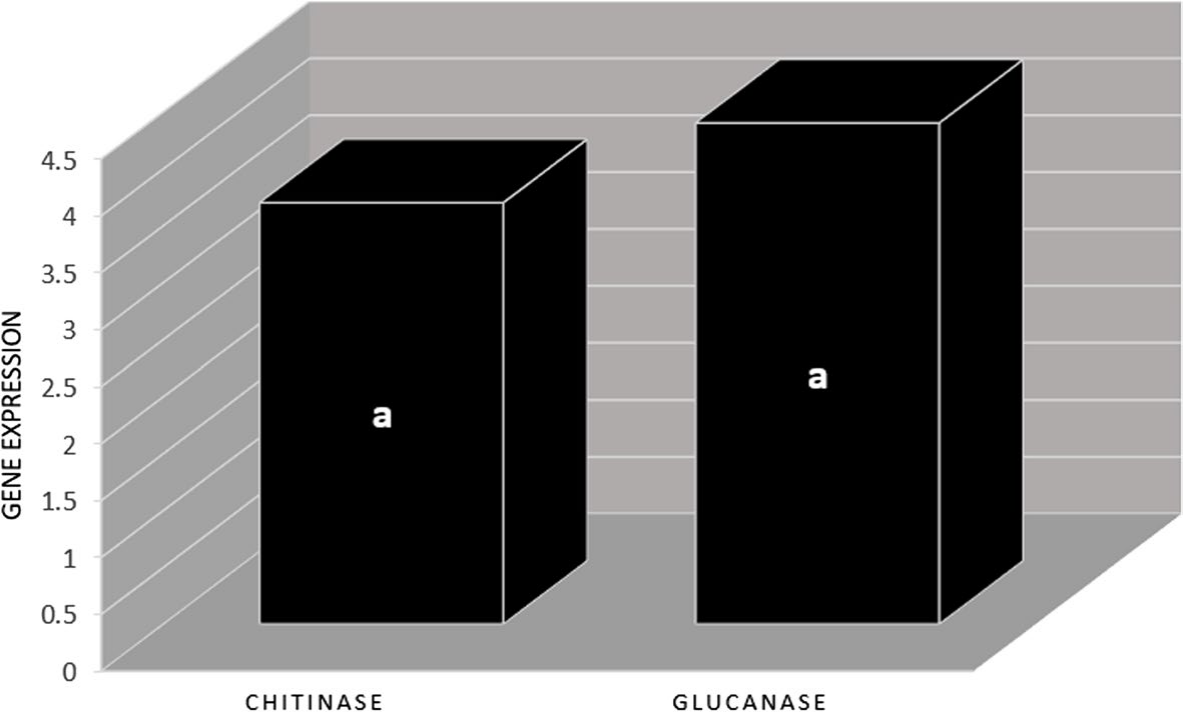
Comparing the expression of chitinase and glucanase genes in transgenic and control wheat plants

Expression of two genes, chitinase and glucanase in plants of transgenic and control groups was compared (three replications). The expression of Chitinase gene in transgenic plants was 3.7 times higher than in the control group. Expression of glucanase in transgenic plants significantly increased (4.4 times) compared to the control group. There was no significant differences between the expression of chitinase and glucanase genes, and these two genes had relatively the same expression levels (Fig. 9).

## Discussion

Fusarium infections can significantly reduce grain productivity and contaminate them with mycotoxins, which can have negative impacts on human and animal health [24]. To prevent colonization of these fungi, plant cells by activate their defense mechanisms by producing pathogenesis-related (PR) proteins [25]. These proteins are often pathogen-specific and have an essential role for inducing systemic acquired resistance in plants [26]. PR proteins are accumulated not only locally in infection sites, but also in normal tissues to induce the resistance against subsequent infection [27]. There are 16 different PRs according to their molecular and physiological properties [28]. The PR1, PR-2 (β-1,3-glucanase), PR-3 (chitinase), PR-4, and PR-5 have been identified as the most important PR proteins in spikes of wheat plants during *F. graminearum* infection [15, 29, 30].

Many studies have shown that overexpression of defense response genes in different transgenic plants such as rice [31], wheat [15], ryegrass [32], tobacco [33], soybean [34], tomato [35, 36], cotton [37], peanut [38], banana [39], finger millet [40], Milk thistle [41], *Aegilops tauschii* [42] and melon [43] increased resistance to different fungal diseases [29]. Overexpression of a transgenic chitinase may enhance the resistance to fungal pathogens at both direct and indirect levels [44]. At the direct level, it enzymatically breaks down chitin found in growing hyphae, whereas at the indirect level, it triggers the release of chitin oligomers, which can serve as elicitors, inducing plant defense mechanisms [45]. Different studies have shown the significant role of β-1,3-glucanase in plant physiology, particularly in its defense against pathogens [27, 46, 47]. It was reported that this gene had higher expression in barley plants grown under organic treatments compared to those in conventional treatments that received pesticides [48]. This enzyme hydrolyze β-1,3-glucans, which are essential components found in the cell walls of different fungal pathogens, weakening the pathogens’ structural integrity, and causing their lysis and death [49–51]. In wheat, antifungal function of glucanase has been thoroughly revealed [15, 52, 53].

In the current study, two chitinase and glucanase genes were transferred to five different Iranian wheat cultivars. To reduce the risk of gene loss through homologous recombination between similar regulatory elements, we employed a construct where these two genes were placed in the opposite directions on the vector. Each gene was expressed individually with its own promoter and terminator. When similar regulatory elements are placed in the same direction, homologous recombination occur between them, resulting in the excision of the DNA segment containing the gene. In contrast, placing these regulatory elements in opposite direction leads to homologous recombination causing an inversion of the DNA segment, rather than the deletion of the gene [54–56]. This construction can lead to an increased stability of transgenes in the next plant generation.

In the current study, we isolated the chitinase from bean and the glucanase from barley. In bean, the primary chitinase activity is linked to a basic, 30-kD protein located in the vacuolar compartment [57, 58]. In a study conducted by Mauch et al. [59], a basic chitinase isolated from bean displayed a strong antifungal effect when tested in vitro. Attia et al. [60] further validated the positive effect of bean chitinase in controlling fungal infections. Mackintosh et al. [15] genetically modified the wheat cultivar (Bobwhite) by introducing transgenes encoding barley β-1,3-glucanase. Balasubramanian et al. [27] conducted a study where they created transgenic pea plants individually by introducing a barley β-1,3-glucanase gene.

Severl studies showed the effectiveness of upregulating chitinase and glucanase gene expression in enhancing plant resistance to fungi.

Zhu et al. [61] were constructed two engineered strains, *Phomopsis liquidambaris* OE-Chi and IN-Chi, through plasmid transformation and chitinase integration into the genome, respectively. Their findings revealed that colonization of the OE-Chi strain in wheat had superior effects compared to colonization of the IN-Chi strain, alleviating the inhibition of wheat growth induced by *F. graminearum*. Raji et al. [62] induced multiple fungal diseases in *Cucumis melo* by co-transformation of different pathogenesis-related (PR) genes. Enzymatic activity assays demonstrated elevated chitinase and b-1,3-glucanase activity in transgenic lines compared to wild-type plants. In-vitro and in-vivo bioassay tests further confirmed increased resistance to fungal diseases in transgenic lines. Taif et al. [63] reported that β-1,3-glucanase gene from *Panax notoginseng* confers resistance in tobacco against *Fusarium solani*. This study indicated the significance of PnGlu1 as a crucial defense gene in response to *F. solani*.

In our study, different regeneration responses were shown among different cultivars, therefore, we conducted this experiment by transforming five distinct wheat cultivars. The assessment of regeneration traits, including shooting and rooting, in both spring and winter wheat cultivars indicated that spring cultivars displayed superior responses compared to winter cultivars. Among the spring cultivars, the A-Line cultivar showed the highest percentage of shooting and rooting (10.2% 11.7%, respectively), followed by Moghan (2.7% shooting and 3.1% rooting) and Arta (1.4% shooting and 2.8% rooting). Among winter cultivars, only the Gascogen cultivar showed shoot formation (0.8%) and no root formation was observed in both cultivars. Similarly, it was shown that the genotype significantly affects the potential of callus induction, the type of callus formed and regeneration of plantlets [64]. In addition, investigating the protein concentration produced by the transgenic plant in response to Fusarium contamination is important. The evaluation of two different protein extract concentrations (100µg and 200µg) showed that the higher concentration had a more pronounced effect on controlling the fungal infections. Our findings were consistent with the results reported by Toufiq et al. [65], who showed that the purified recombinant chitinase protein significantly inhibited essential phytopathogenic fungi (particularly at concentrations of 80μg and 200μg) when compared to the control. Altogether, through improving the above-mentioned conditions, we successfully generated transgenic wheat plants that demonstrated resistance against Fusarium.

## Conclusion

Fusarium causes significant damage to agricultural products every year, with wheat being particularly susceptible. To cope with it, plants naturally synthesize pathogenesis-related (PR) proteins. In our study, two PR proteins, chitinase and glucanase, were used to develop Fusarium-resistant wheat. Using the Biolistic PDS-1000/He Particle Delivery System, we introduced these two genes into five different cultivars of Iranian wheat of which only two cultivars (A-Line and Moghan) successfully incorporated these genes. PCR and Southern blot analysis confirmed integration of these genes into genome. The bioassay results showed that the transgenic plants were resistant to Fusarium when assessed under in vitro conditions.

### Supplementary Information

Supplementary Material 1.

Supplementary Material 2.

Supplementary Material 3.

Supplementary Material 4.

Supplementary Material 5.

## Data Availability

No datasets were generated or analysed during the current study.

## Footnotes

1 The (-) sign indicates that the two genes are in opposite directions.
