# Supplementary figures and images for: Co-overexpression of chitinase and β-1,3-glucanase significantly enhanced the resistance of Iranian wheat cultivars to Fusarium
Source: BMC Biotechnol. 2024 May 24;24:35. doi: 10.1186/s12896-024-00859-0 (PMC11127306; doi:10.1186/s12896-024-00859-0)

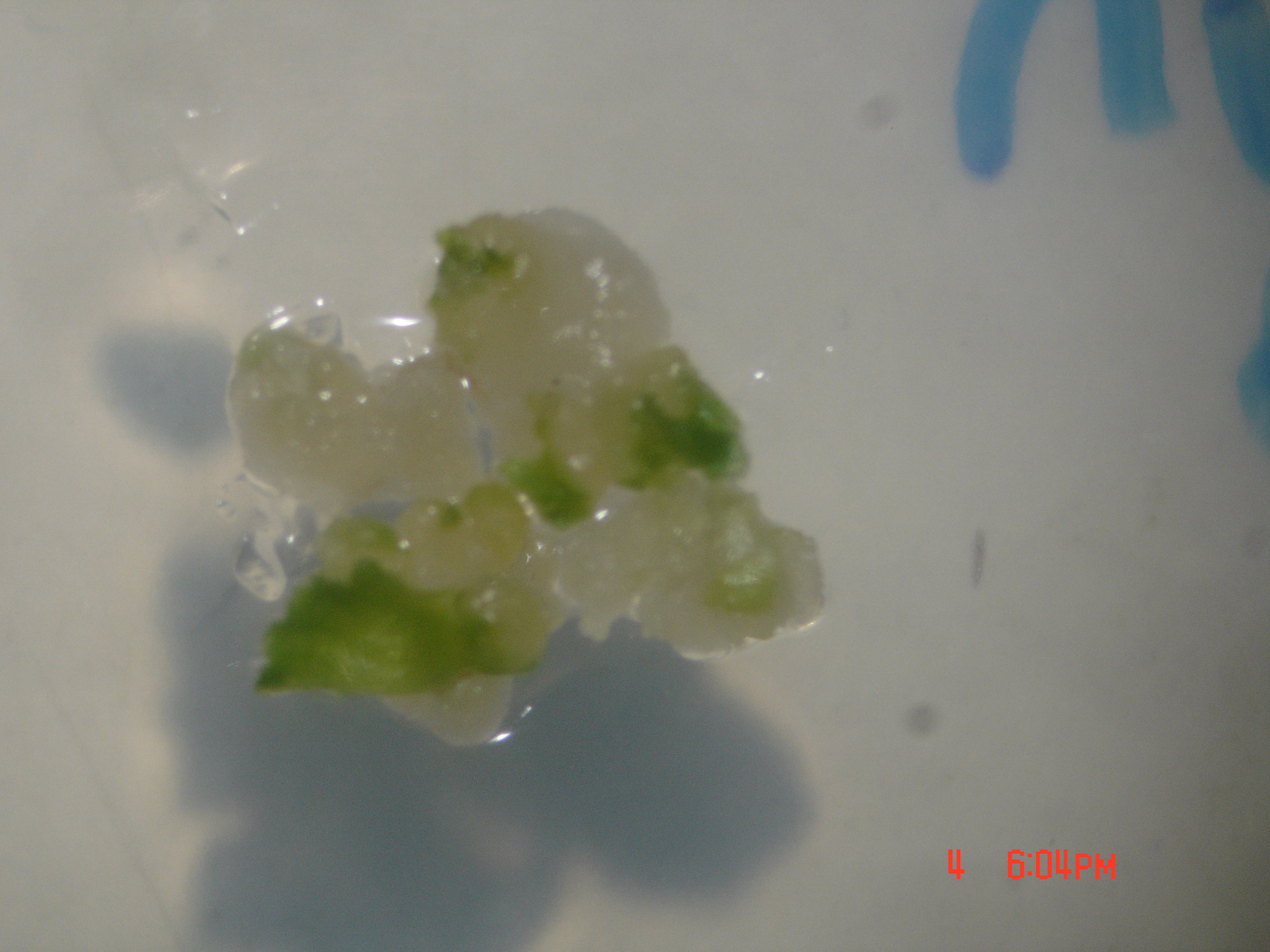

Supplement: Supplementary file 1 — Supplementary Material 1. [file 12896_2024_859_MOESM1_ESM.zip › ax کالوس/DSC00018.JPG]

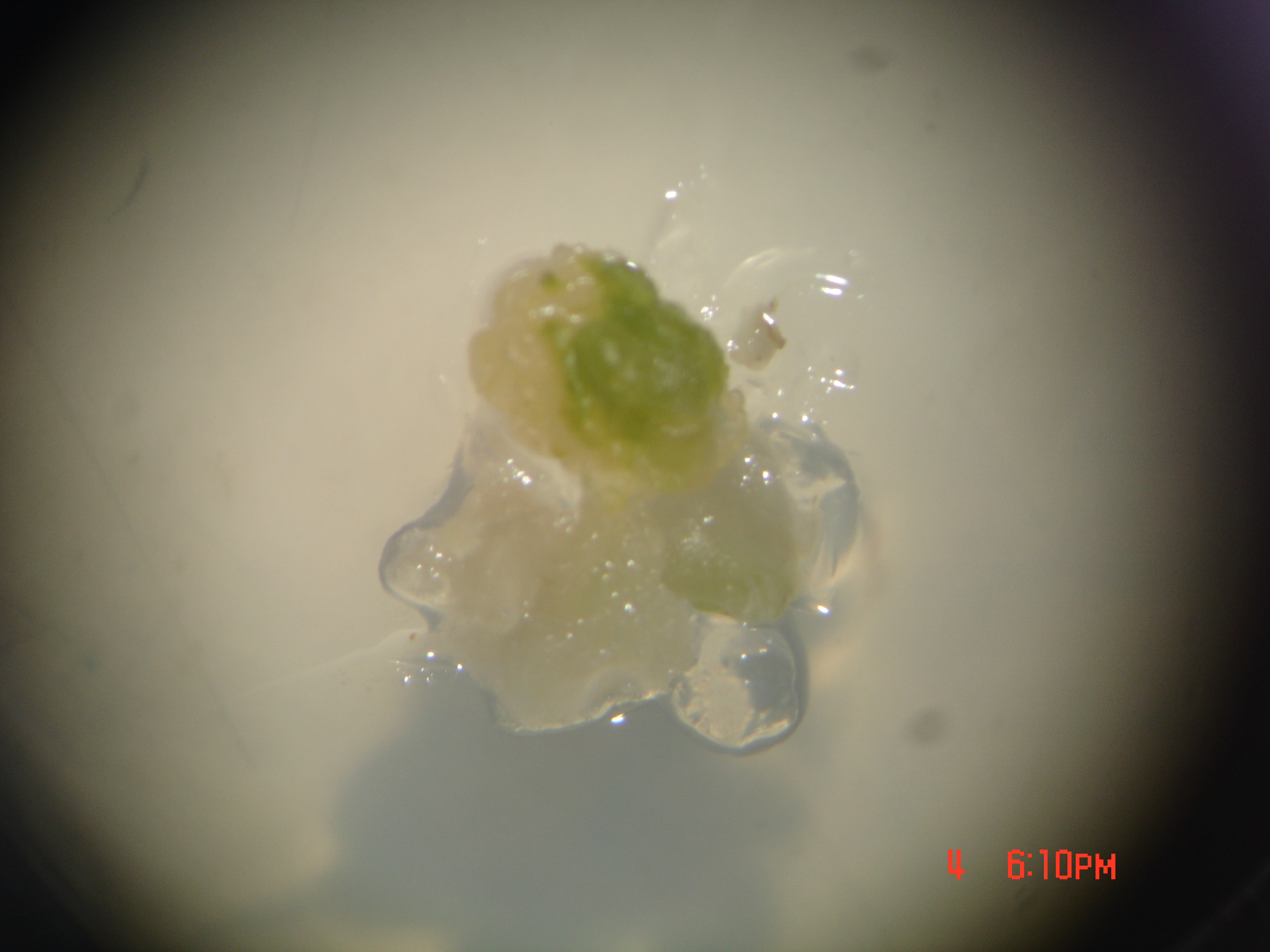

Supplement: Supplementary file 1 — Supplementary Material 1. [file 12896_2024_859_MOESM1_ESM.zip › ax کالوس/DSC00027.JPG]

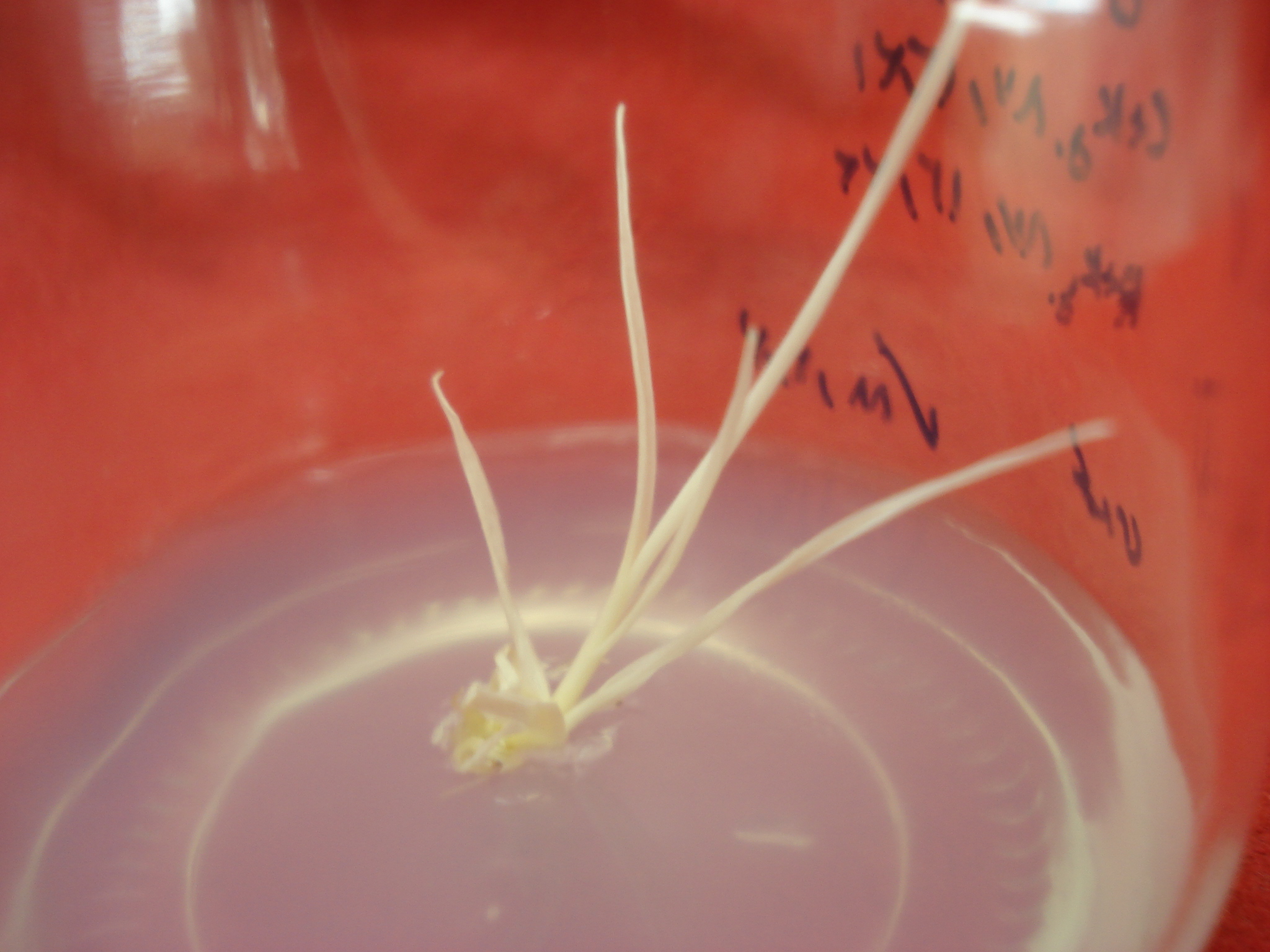

Supplement: Supplementary file 1 — Supplementary Material 1. [file 12896_2024_859_MOESM1_ESM.zip › ax کالوس/DSC08103.JPG]

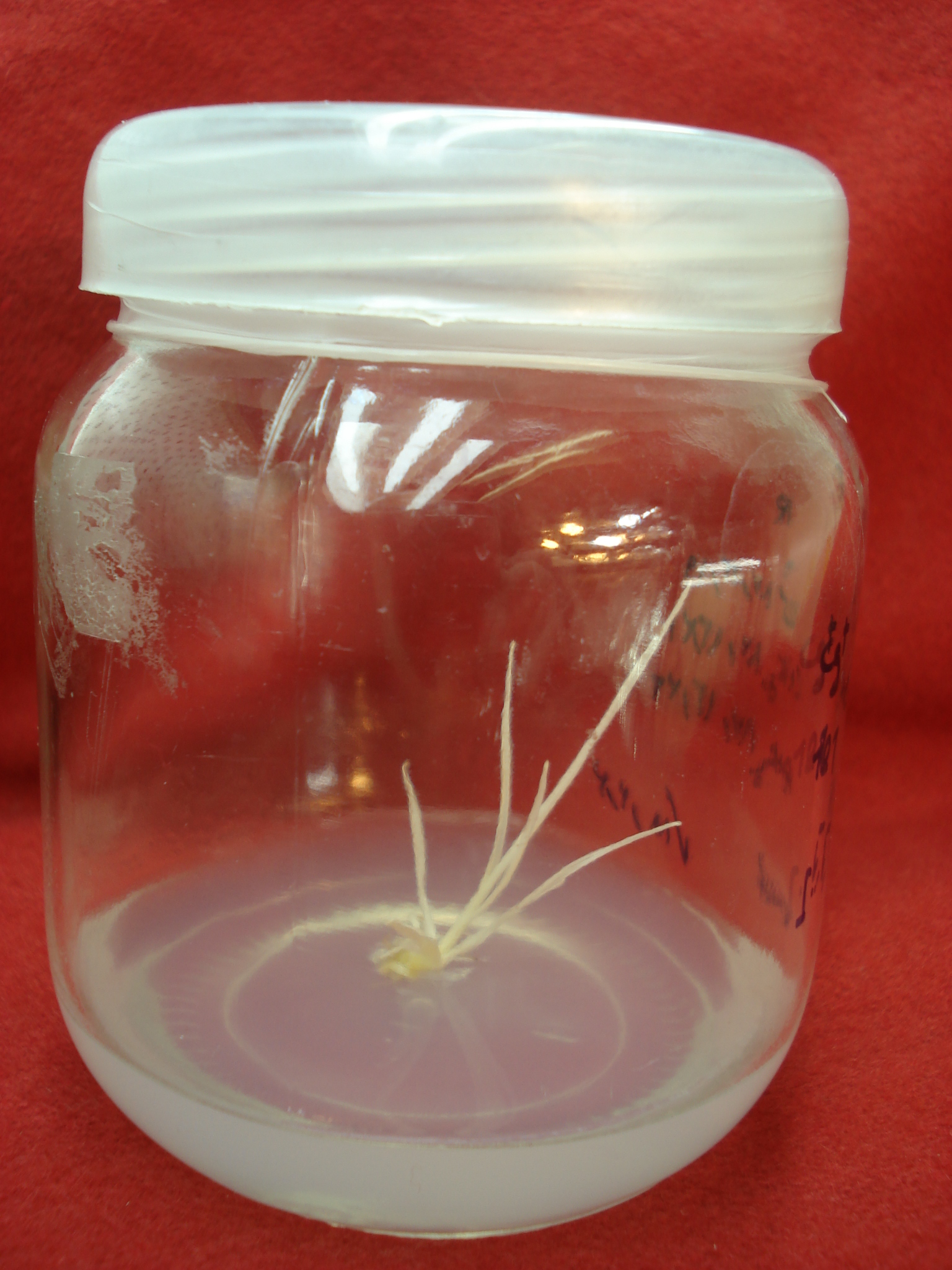

Supplement: Supplementary file 1 — Supplementary Material 1. [file 12896_2024_859_MOESM1_ESM.zip › ax کالوس/DSC08109.JPG]

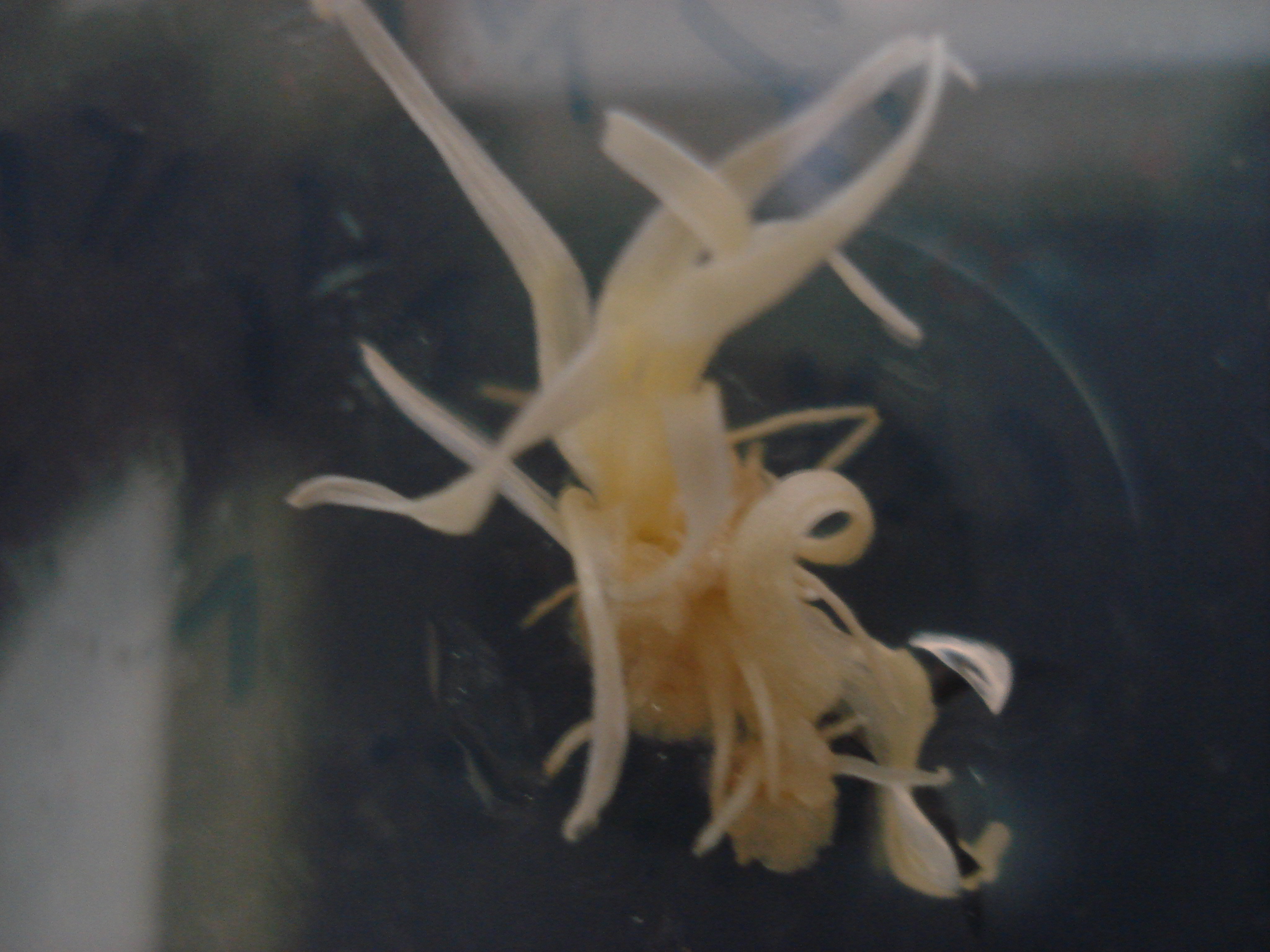

Supplement: Supplementary file 1 — Supplementary Material 1. [file 12896_2024_859_MOESM1_ESM.zip › ax کالوس/DSC08149.JPG]

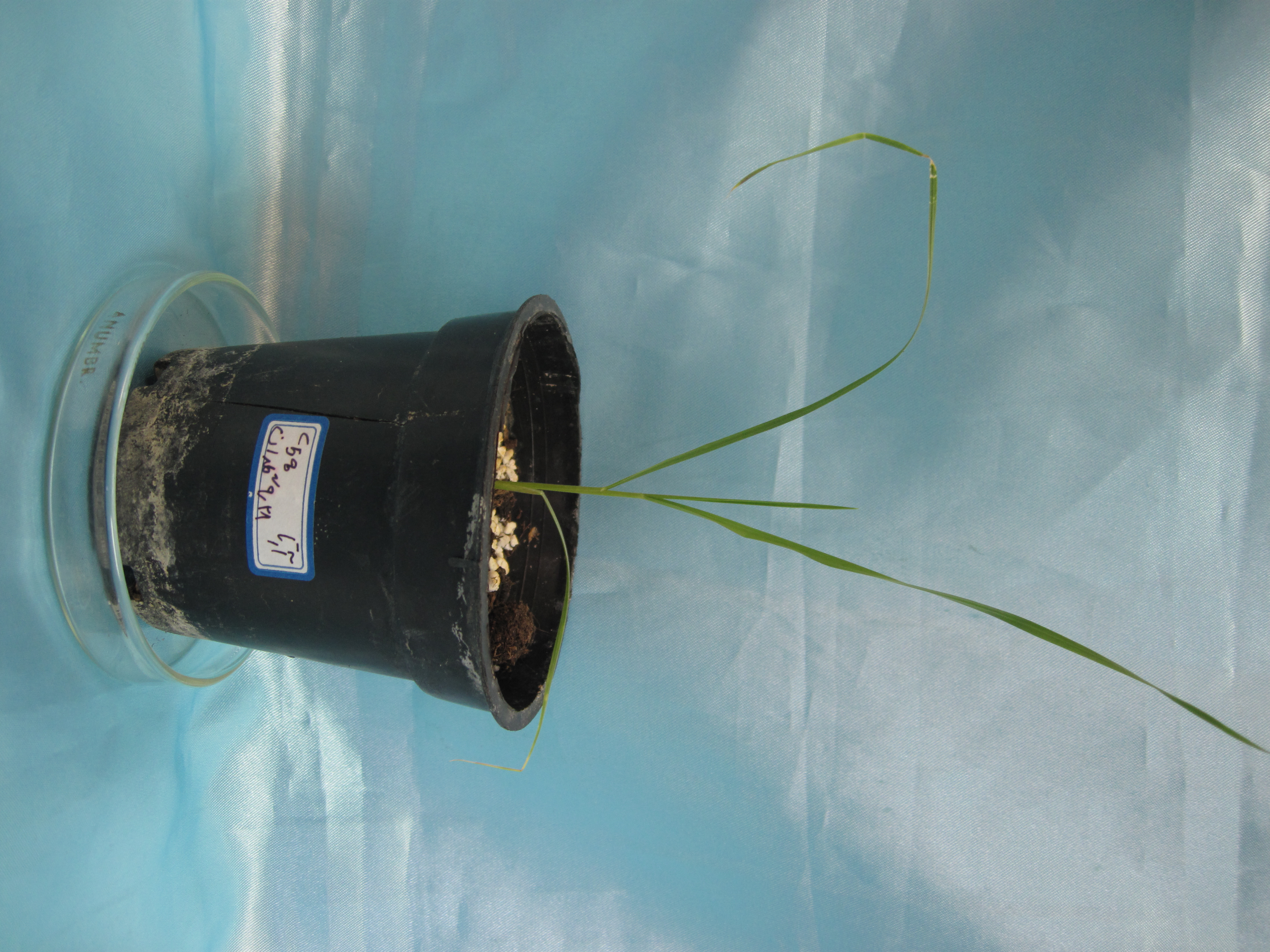

Supplement: Supplementary file 1 — Supplementary Material 1. [file 12896_2024_859_MOESM1_ESM.zip › ax کالوس/IMG_0339.JPG]

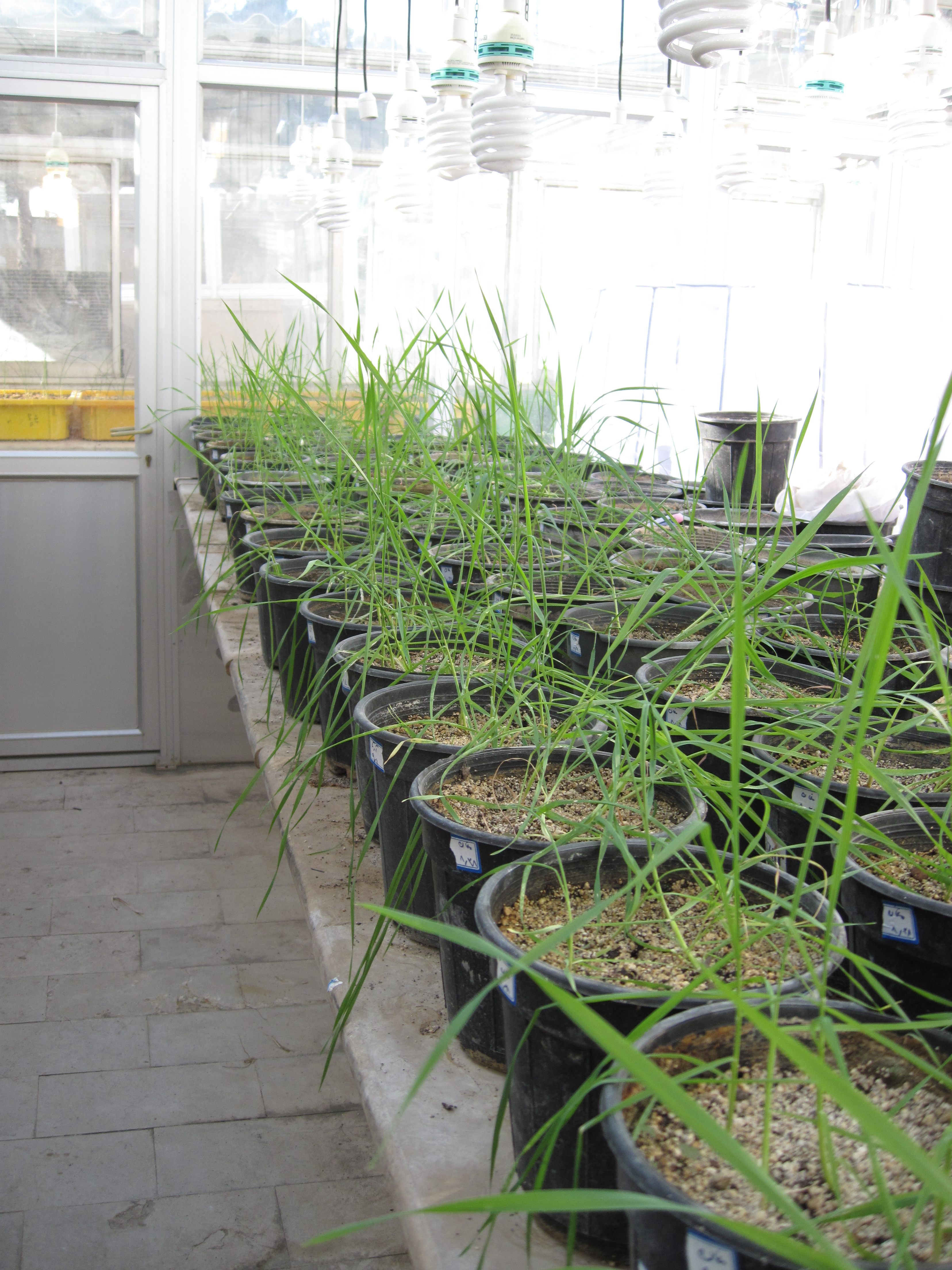

Supplement: Supplementary file 1 — Supplementary Material 1. [file 12896_2024_859_MOESM1_ESM.zip › ax کالوس/IMG_0349.JPG]

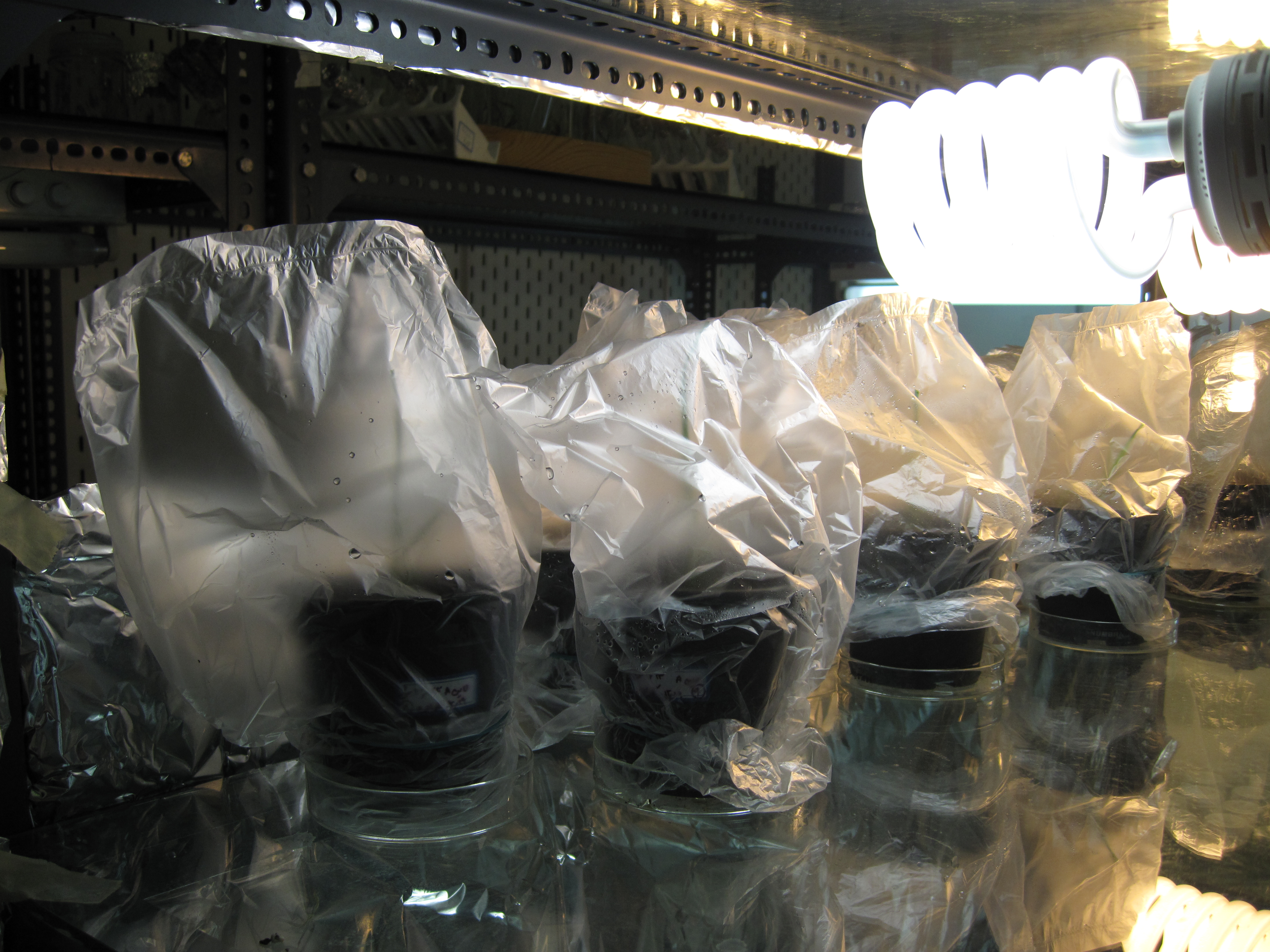

Supplement: Supplementary file 1 — Supplementary Material 1. [file 12896_2024_859_MOESM1_ESM.zip › ax کالوس/IMG_0410.JPG]

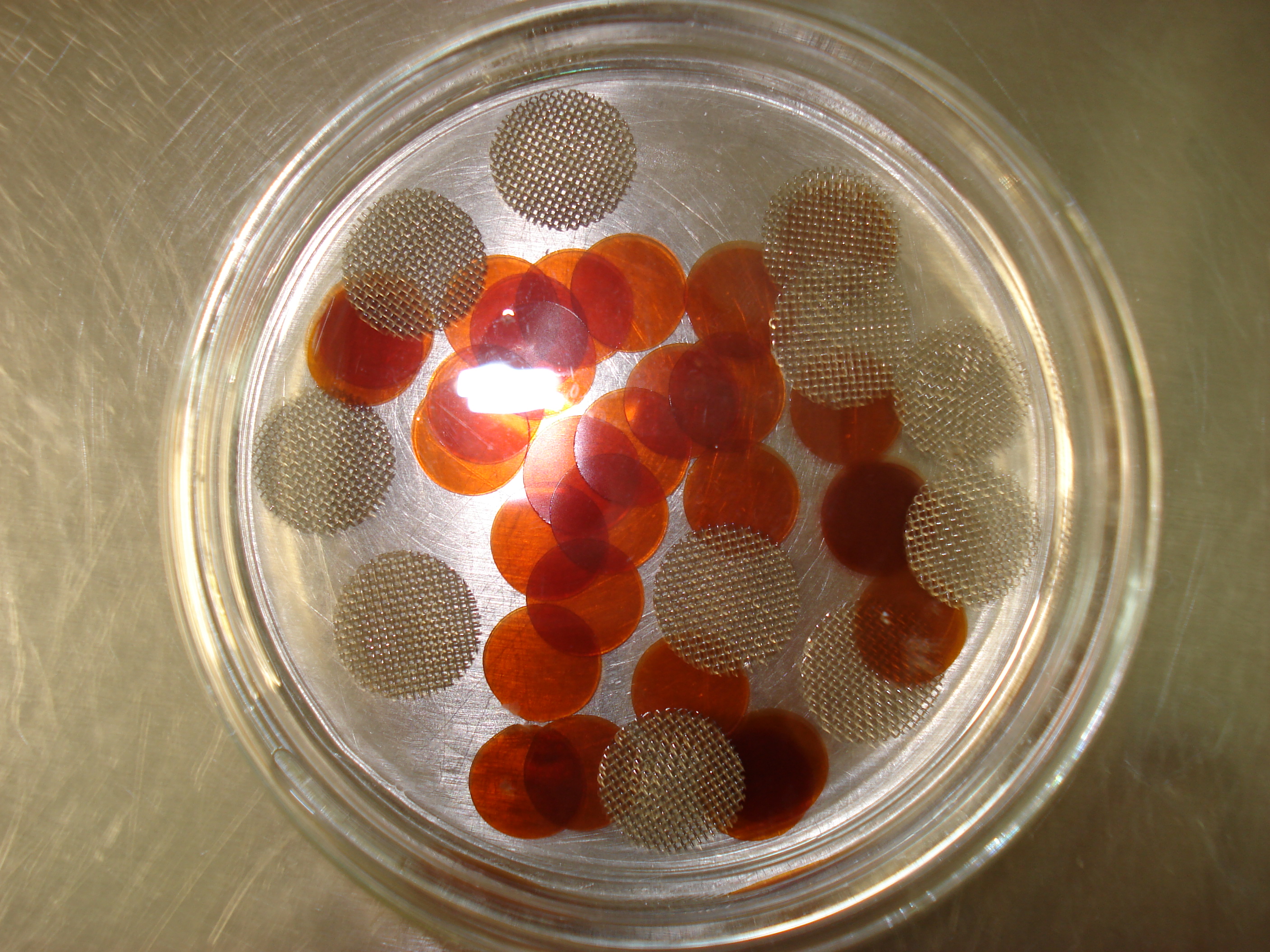

Supplement: Supplementary file 2 — Supplementary Material 2. [file 12896_2024_859_MOESM2_ESM.zip › axجنین/DSC08425.JPG]

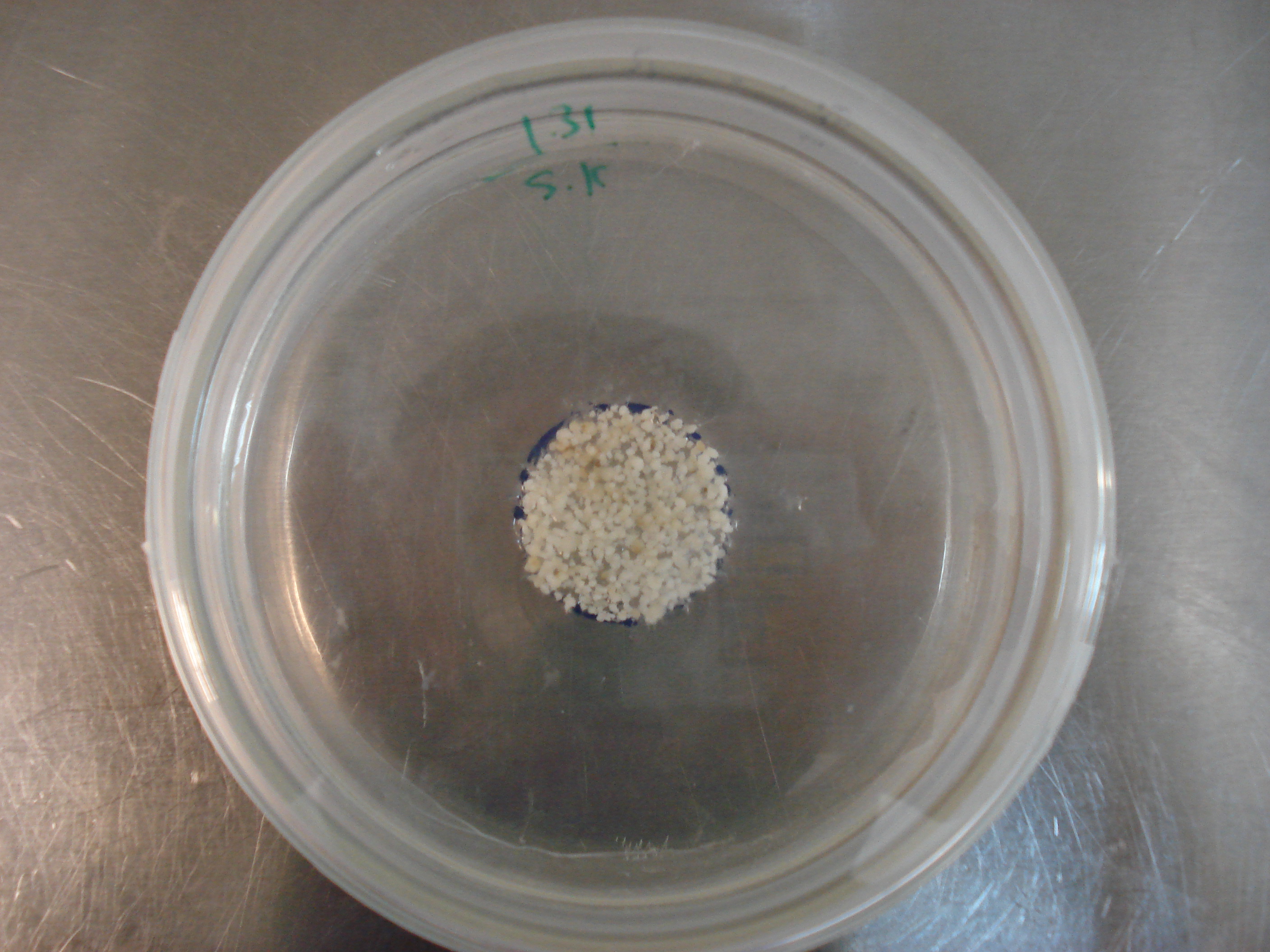

Supplement: Supplementary file 2 — Supplementary Material 2. [file 12896_2024_859_MOESM2_ESM.zip › axجنین/DSC08430.JPG]

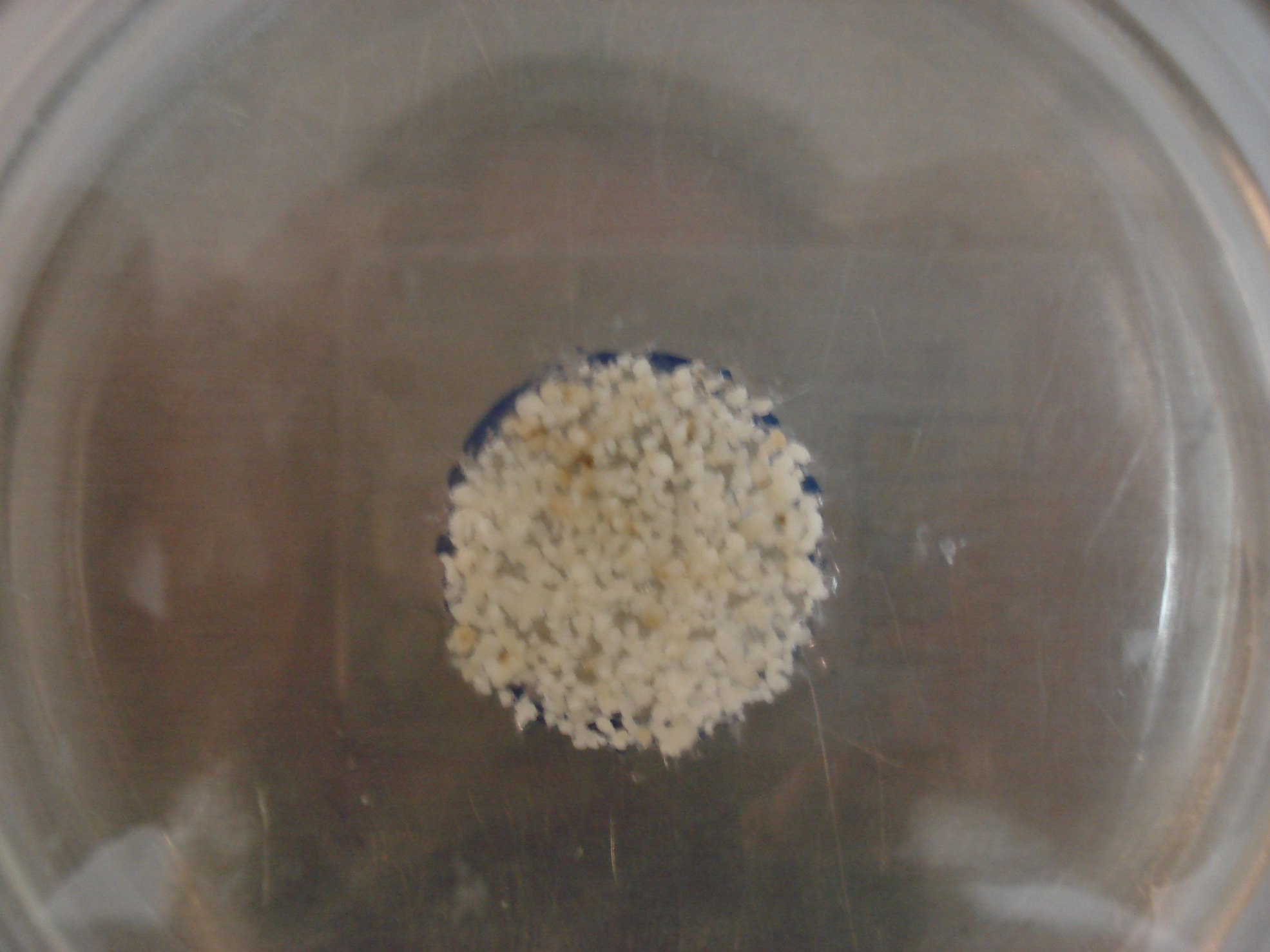

Supplement: Supplementary file 2 — Supplementary Material 2. [file 12896_2024_859_MOESM2_ESM.zip › axجنین/DSC08431.JPG]

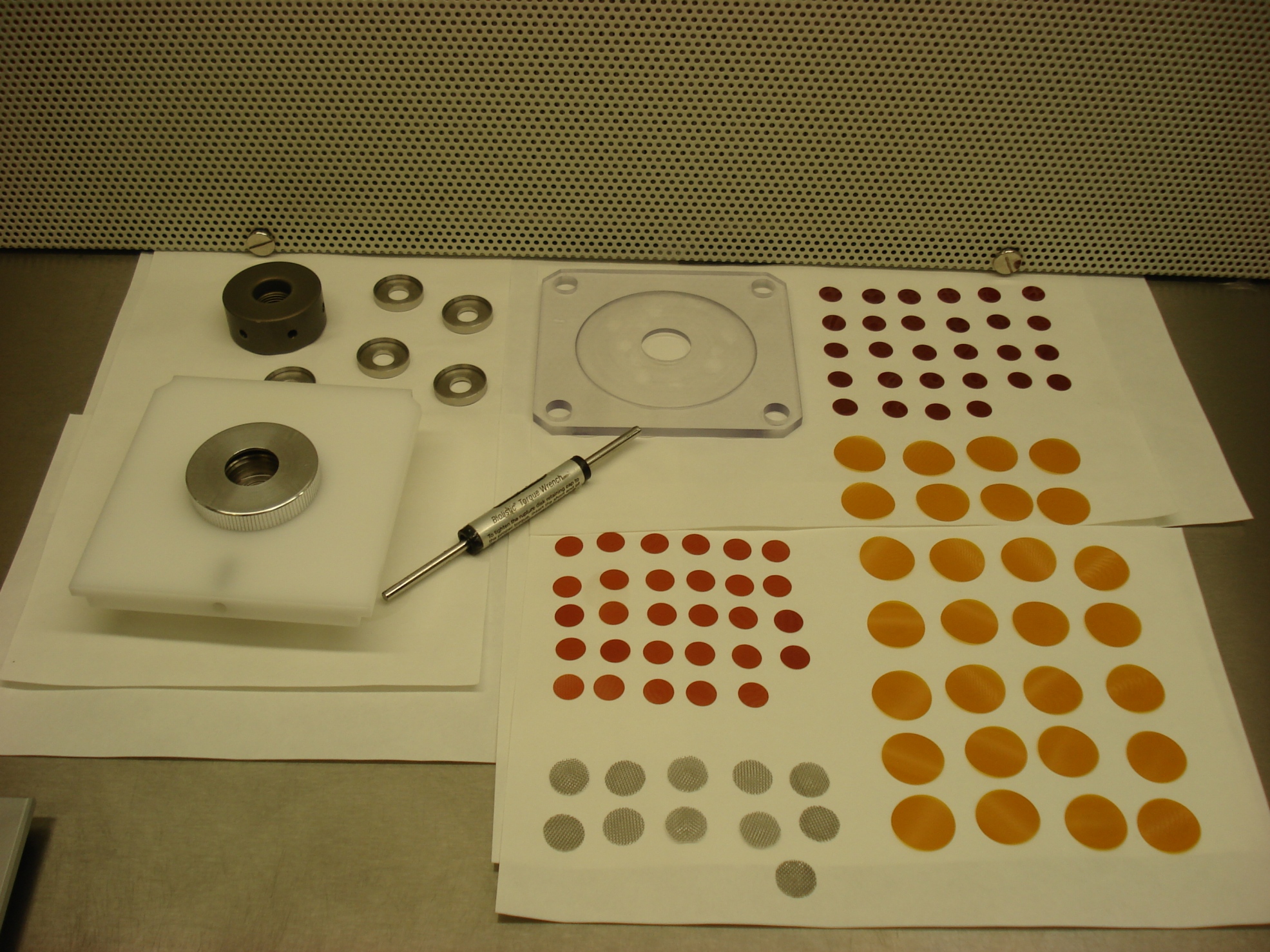

Supplement: Supplementary file 2 — Supplementary Material 2. [file 12896_2024_859_MOESM2_ESM.zip › axجنین/DSC08444.JPG]

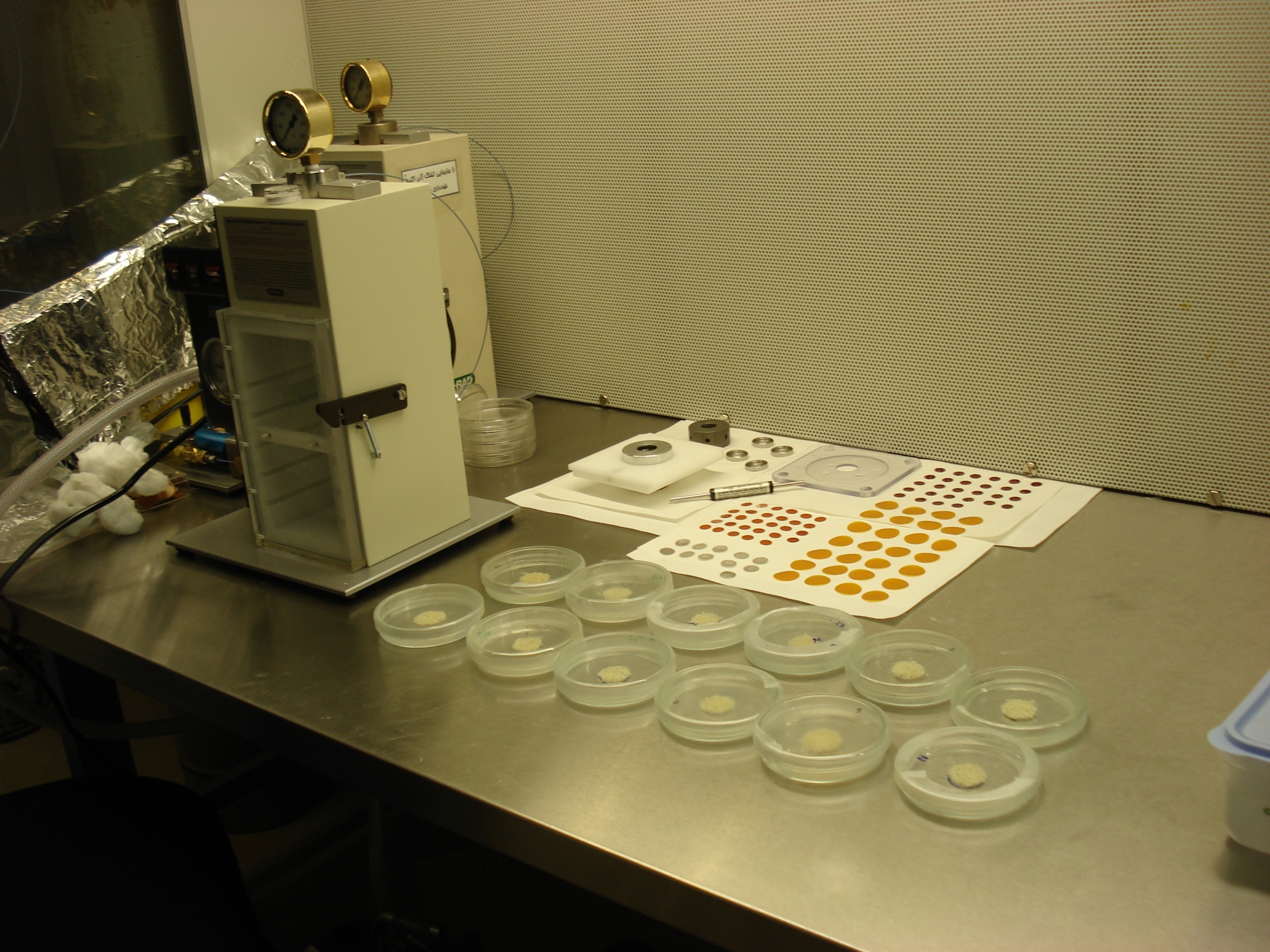

Supplement: Supplementary file 2 — Supplementary Material 2. [file 12896_2024_859_MOESM2_ESM.zip › axجنین/DSC08446.JPG]

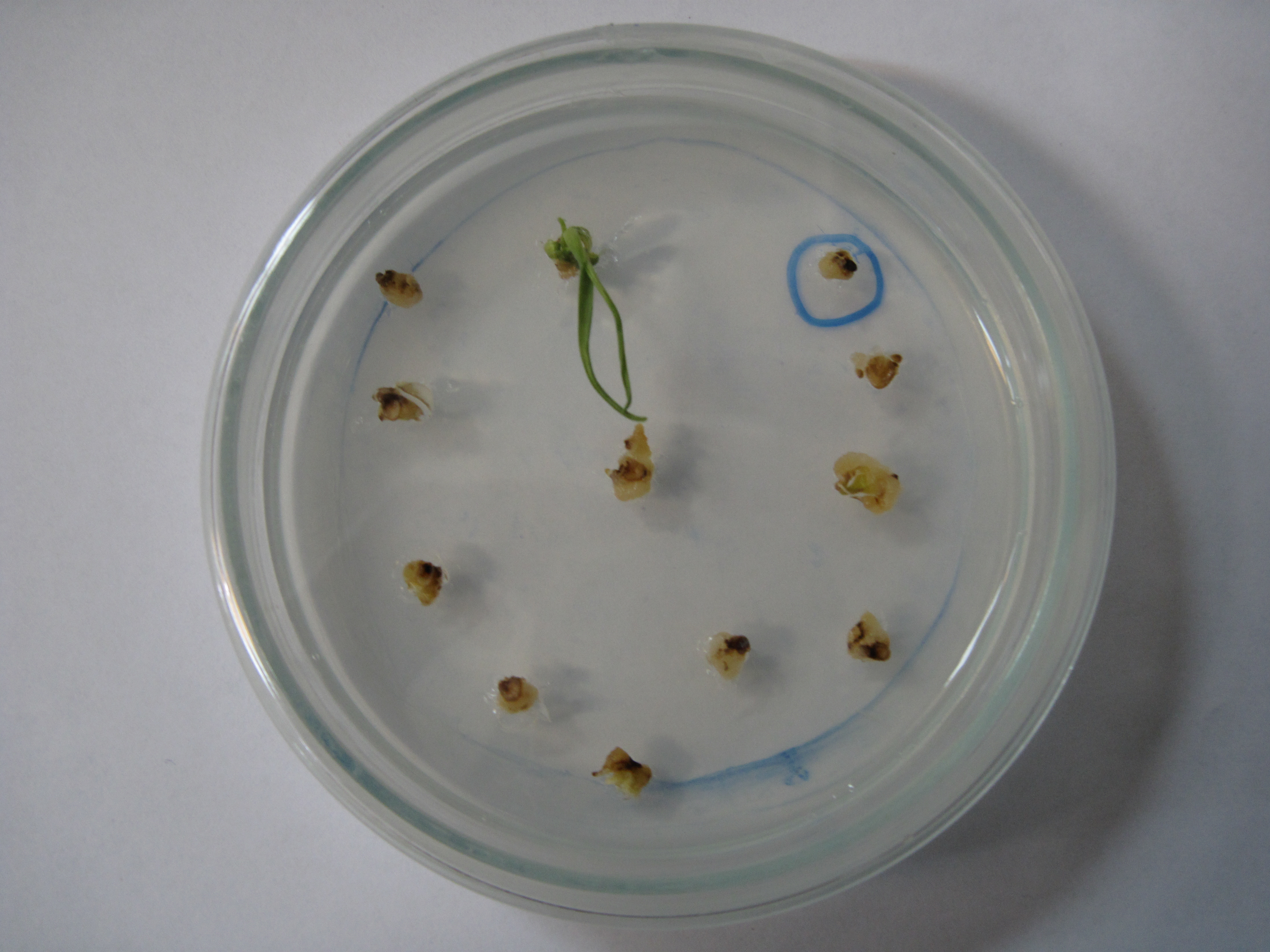

Supplement: Supplementary file 2 — Supplementary Material 2. [file 12896_2024_859_MOESM2_ESM.zip › axجنین/IMG_0256.JPG]

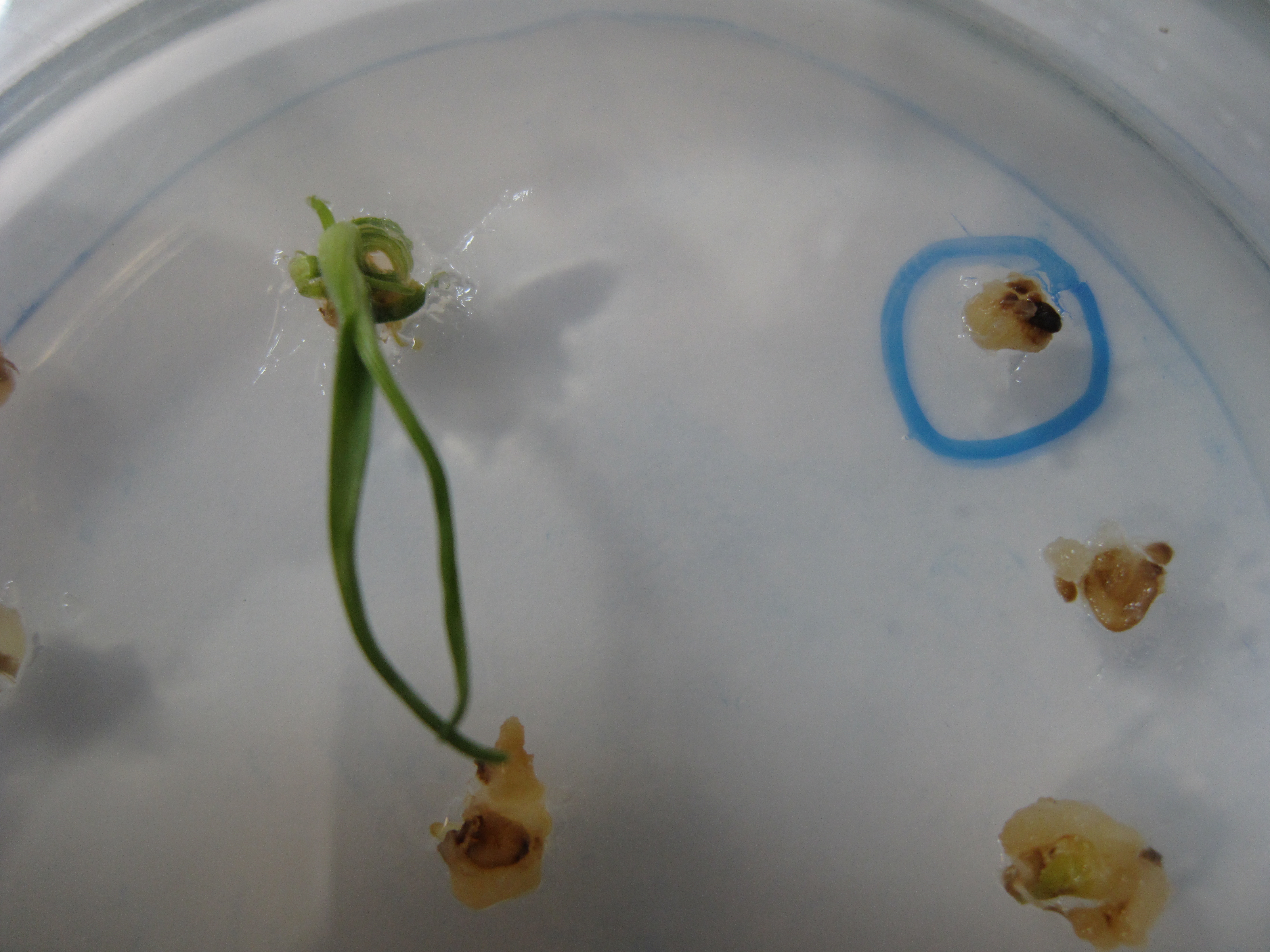

Supplement: Supplementary file 2 — Supplementary Material 2. [file 12896_2024_859_MOESM2_ESM.zip › axجنین/IMG_0258.JPG]

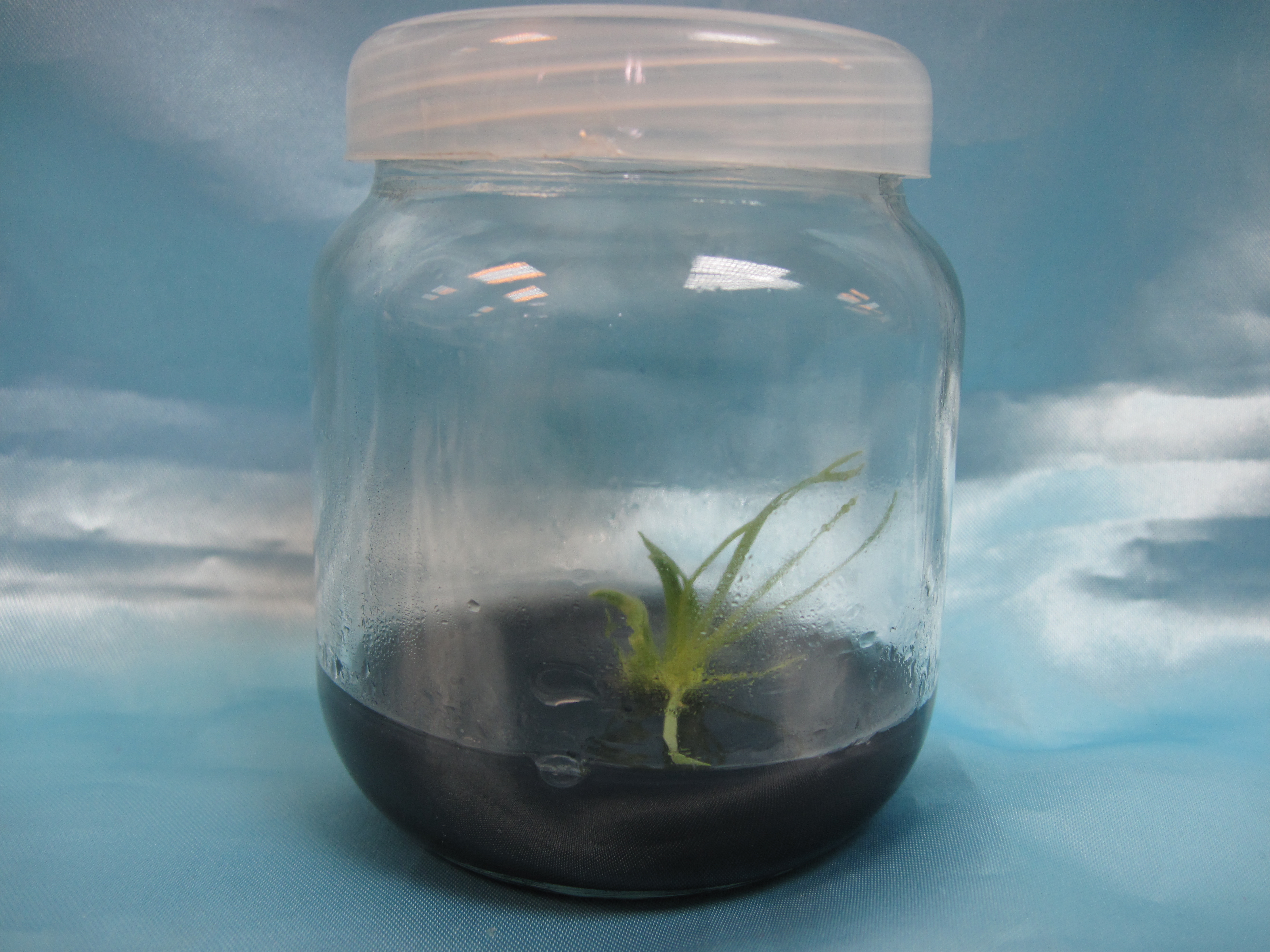

Supplement: Supplementary file 2 — Supplementary Material 2. [file 12896_2024_859_MOESM2_ESM.zip › axجنین/IMG_0336.JPG]

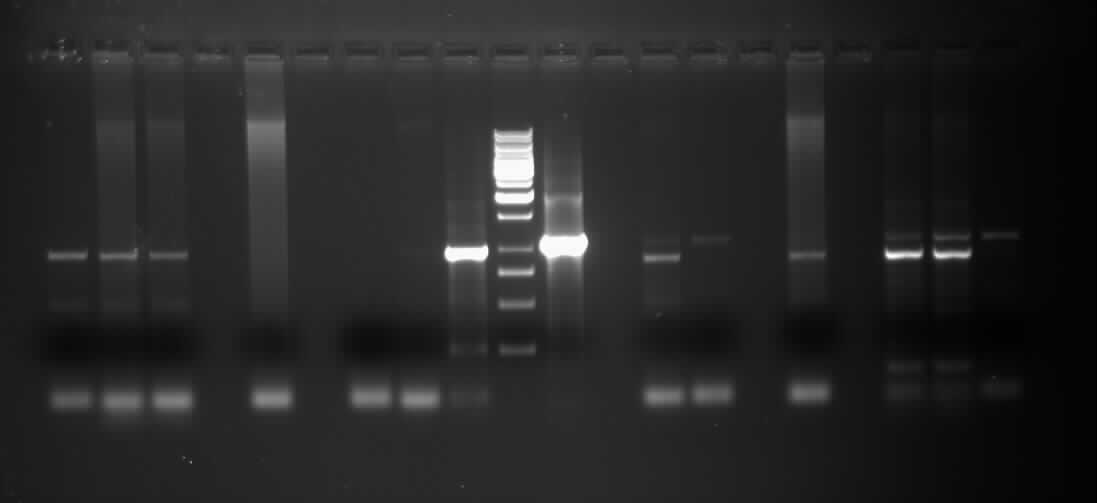

Supplement: Supplementary file 3 — Supplementary Material 3. [file 12896_2024_859_MOESM3_ESM.zip › ax gel/12-11-88 1.jpg]

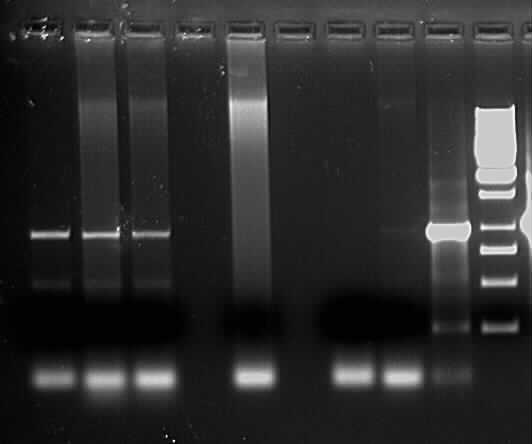

Supplement: Supplementary file 3 — Supplementary Material 3. [file 12896_2024_859_MOESM3_ESM.zip › ax gel/12-11-88 3.jpg]

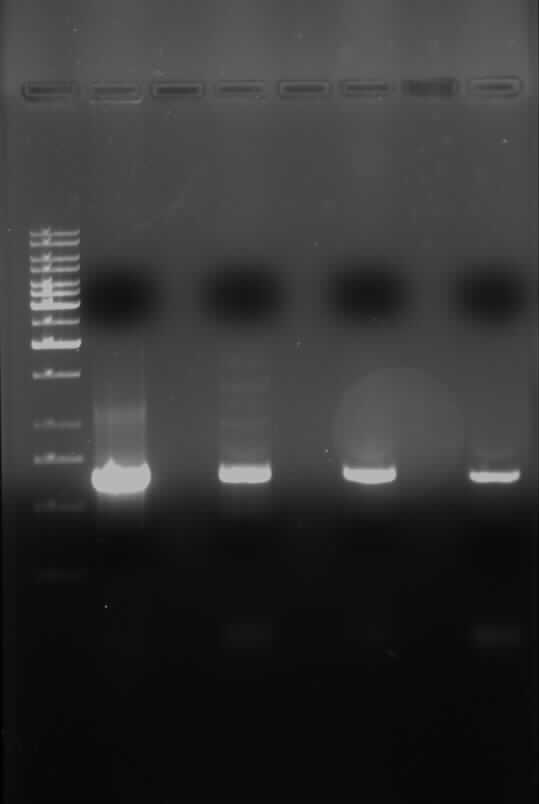

Supplement: Supplementary file 3 — Supplementary Material 3. [file 12896_2024_859_MOESM3_ESM.zip › ax gel/12.6.88-2.jpg]

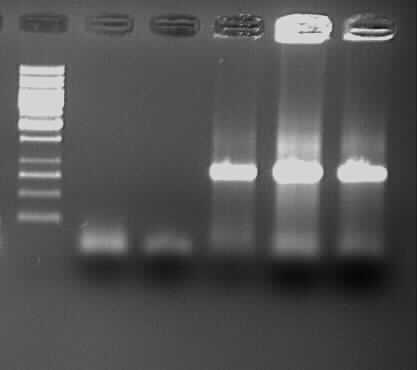

Supplement: Supplementary file 3 — Supplementary Material 3. [file 12896_2024_859_MOESM3_ESM.zip › ax gel/4-12-87.jpg]

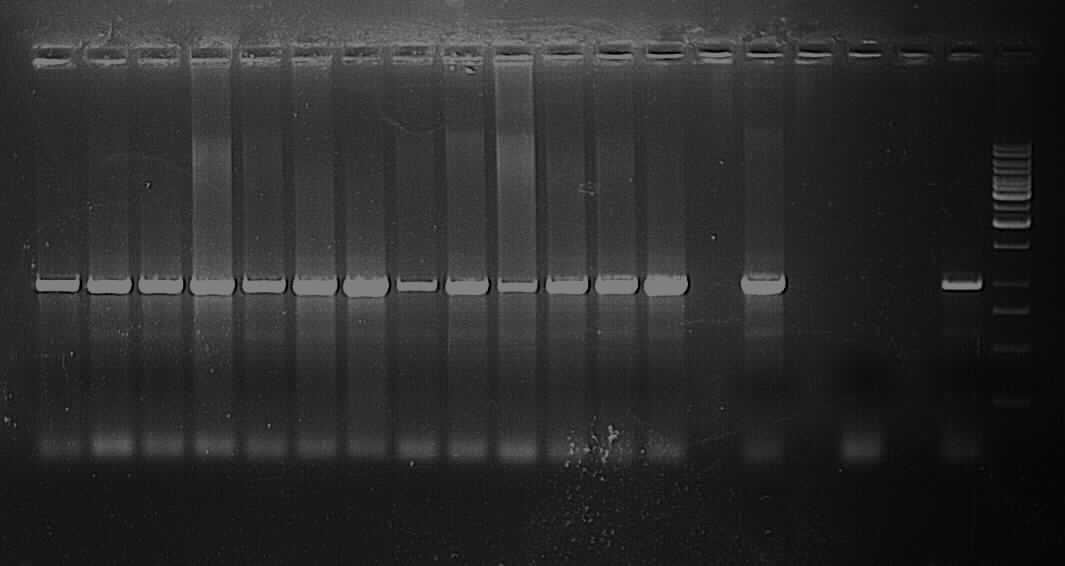

Supplement: Supplementary file 3 — Supplementary Material 3. [file 12896_2024_859_MOESM3_ESM.zip › ax gel/88.12.26-pcr line a.jpg]

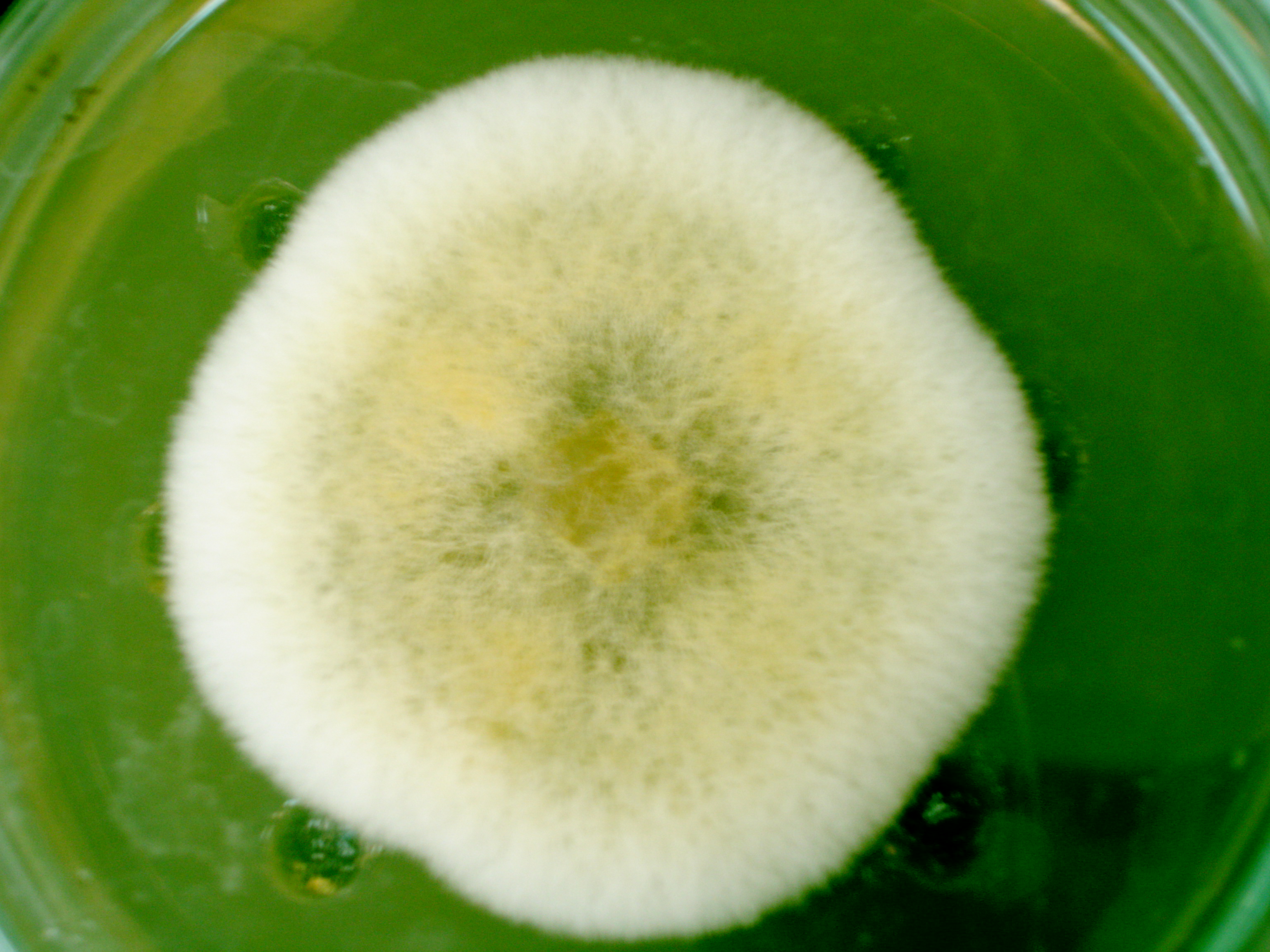

Supplement: Supplementary file 4 — Supplementary Material 4. [file 12896_2024_859_MOESM4_ESM.zip › ax invitroassay/DSC00001.JPG]

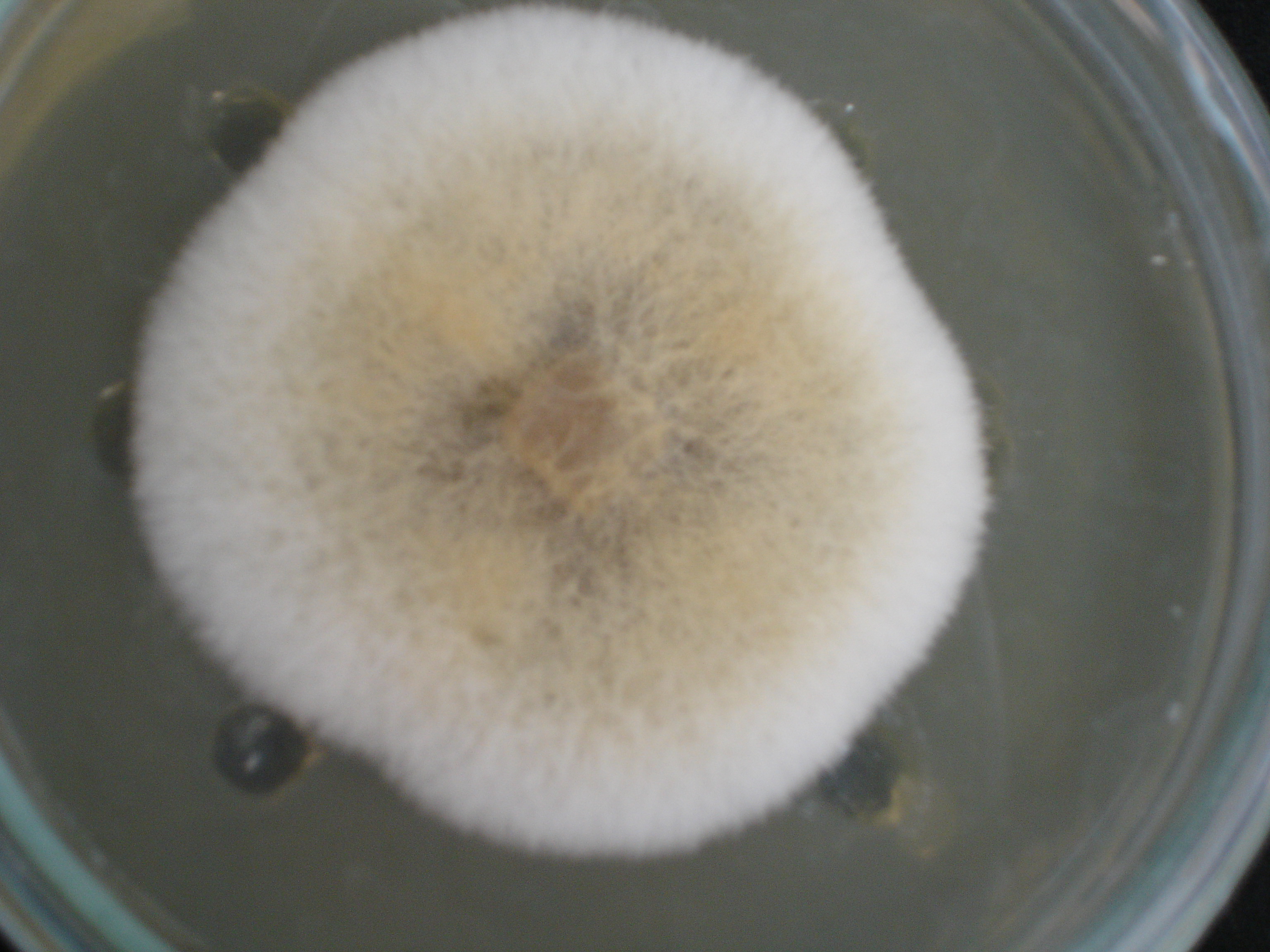

Supplement: Supplementary file 4 — Supplementary Material 4. [file 12896_2024_859_MOESM4_ESM.zip › ax invitroassay/DSC00002.JPG]

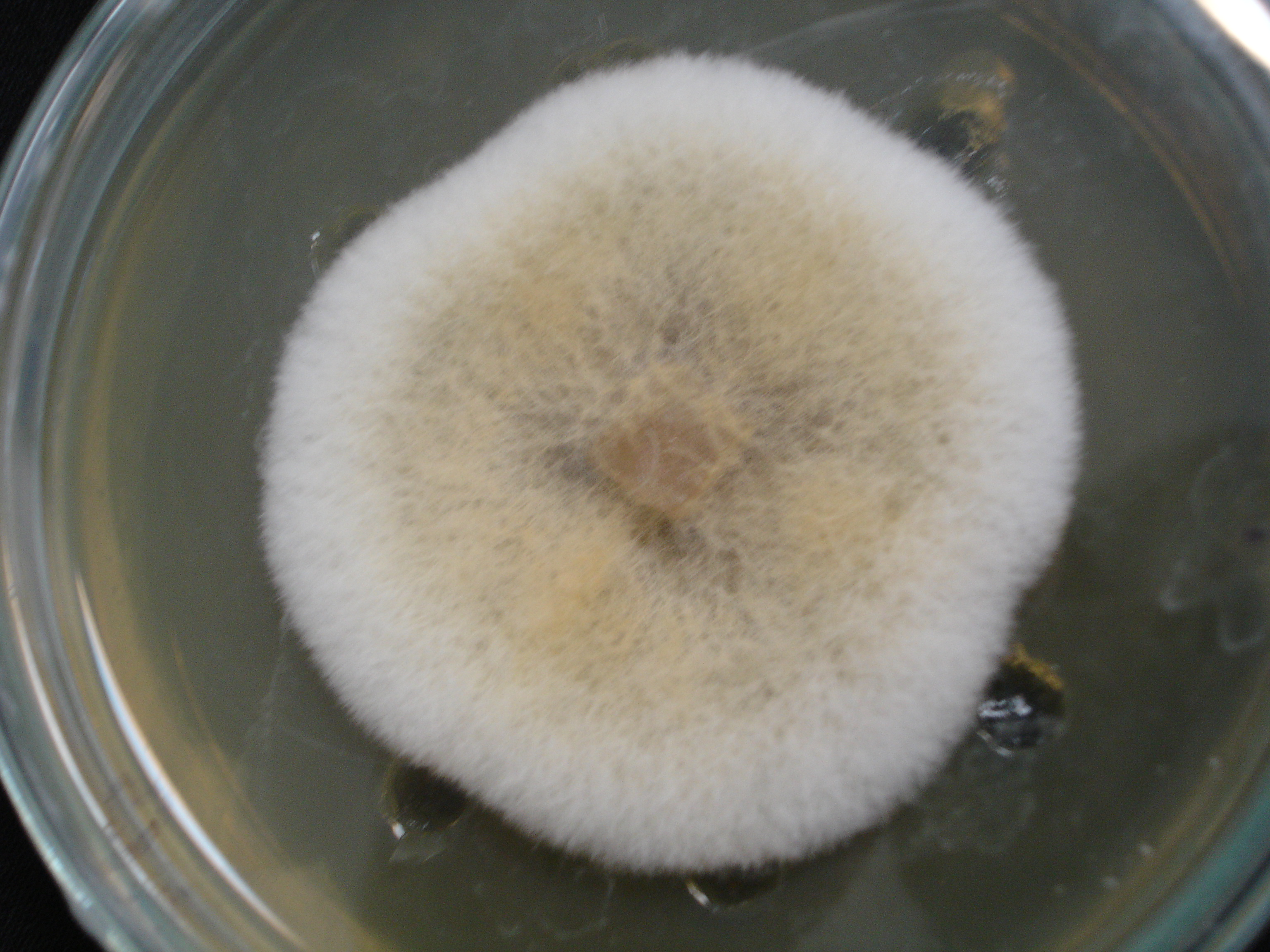

Supplement: Supplementary file 4 — Supplementary Material 4. [file 12896_2024_859_MOESM4_ESM.zip › ax invitroassay/DSC00003.JPG]

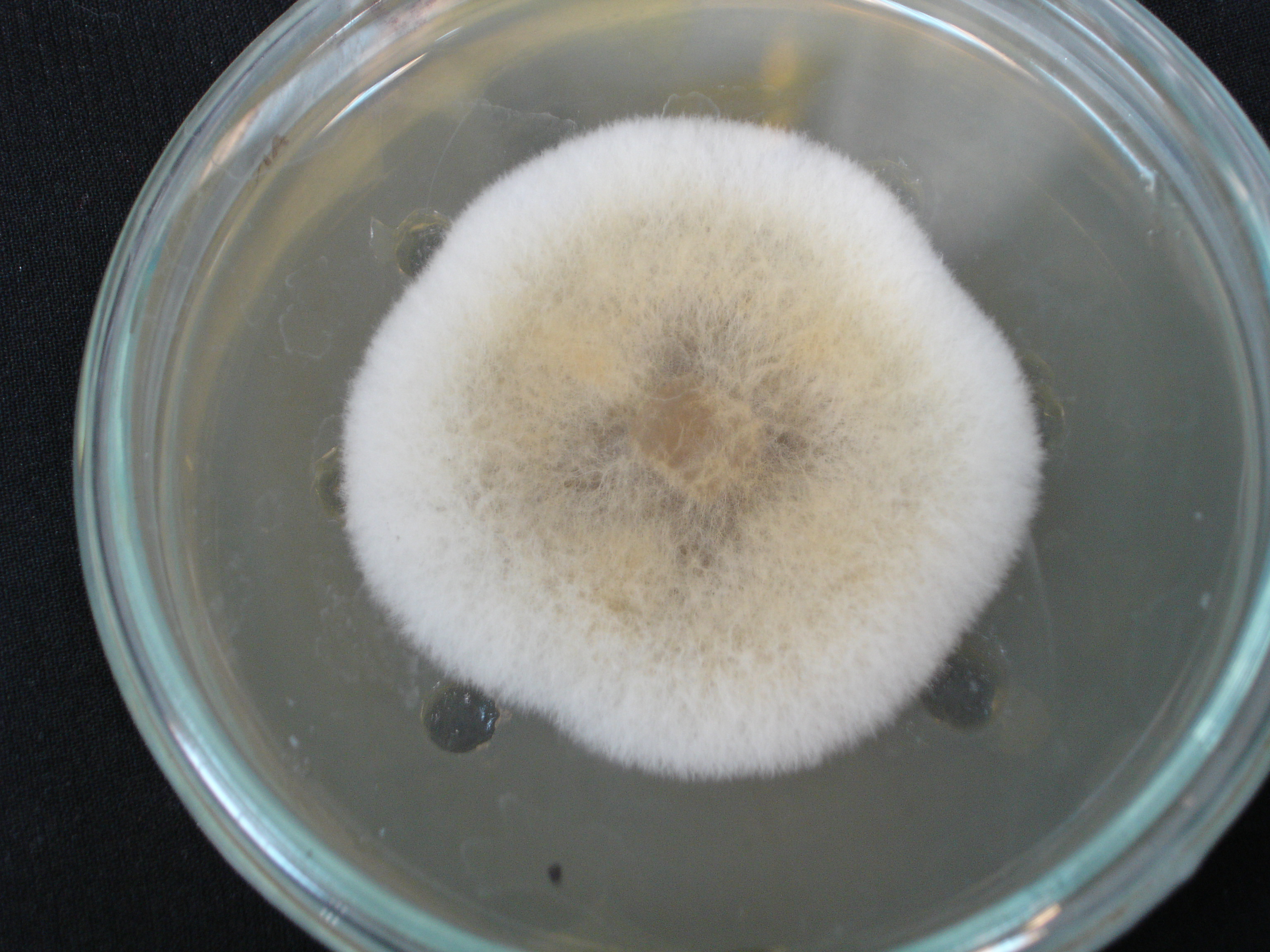

Supplement: Supplementary file 4 — Supplementary Material 4. [file 12896_2024_859_MOESM4_ESM.zip › ax invitroassay/DSC00004.JPG]

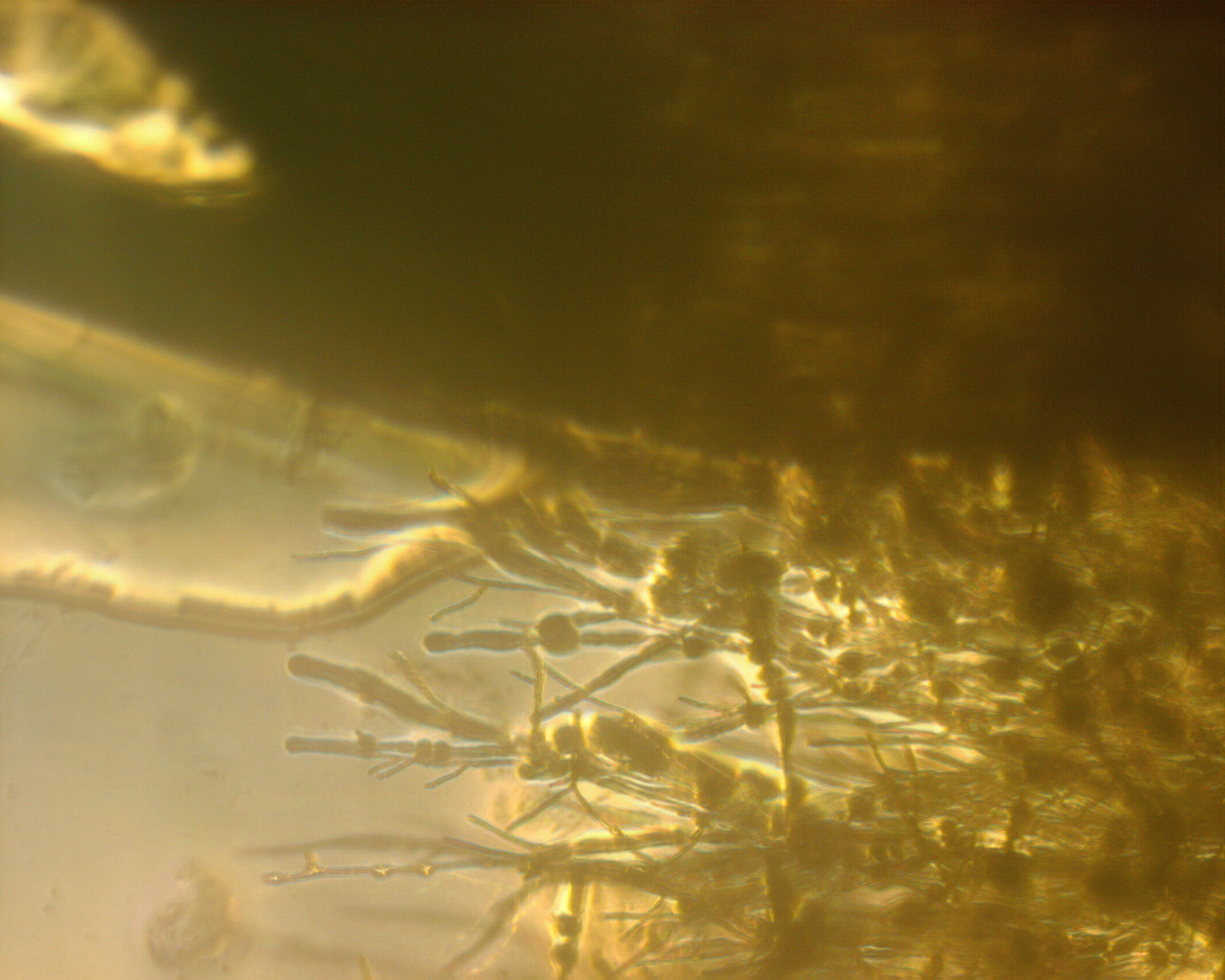

Supplement: Supplementary file 4 — Supplementary Material 4. [file 12896_2024_859_MOESM4_ESM.zip › ax invitroassay/img_bb001.jpg]

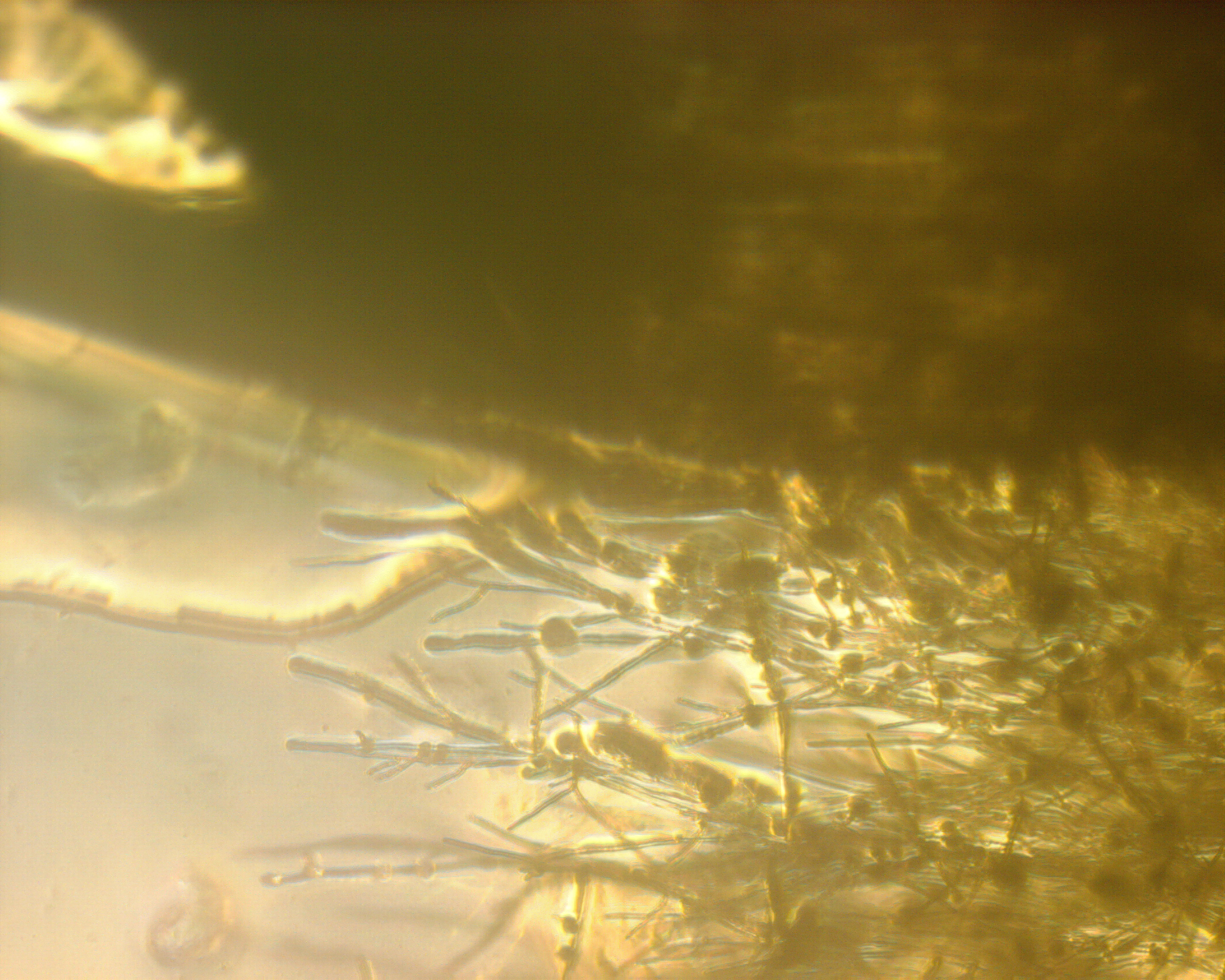

Supplement: Supplementary file 4 — Supplementary Material 4. [file 12896_2024_859_MOESM4_ESM.zip › ax invitroassay/img_bb002.jpg]

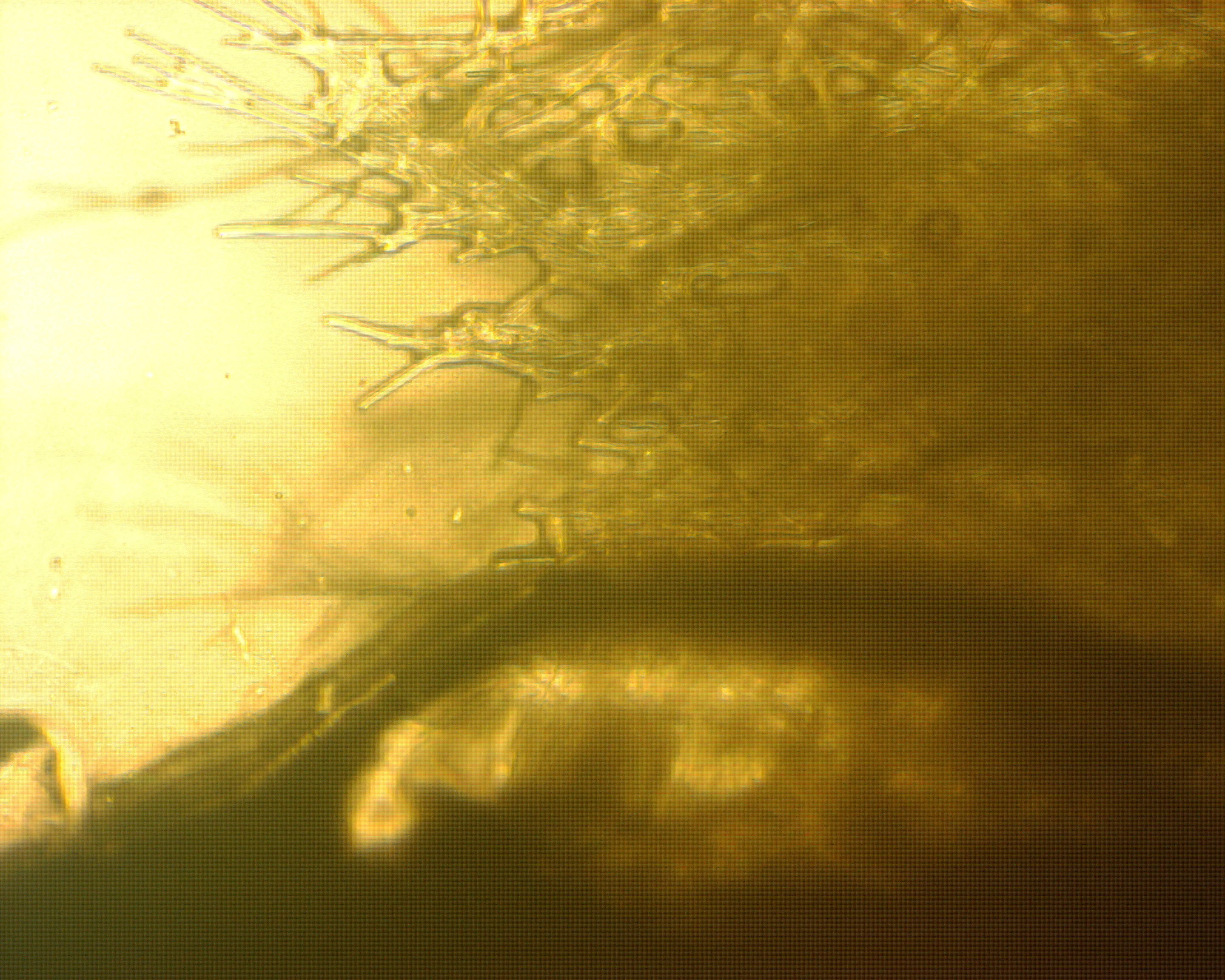

Supplement: Supplementary file 4 — Supplementary Material 4. [file 12896_2024_859_MOESM4_ESM.zip › ax invitroassay/img_shahed001.jpg]

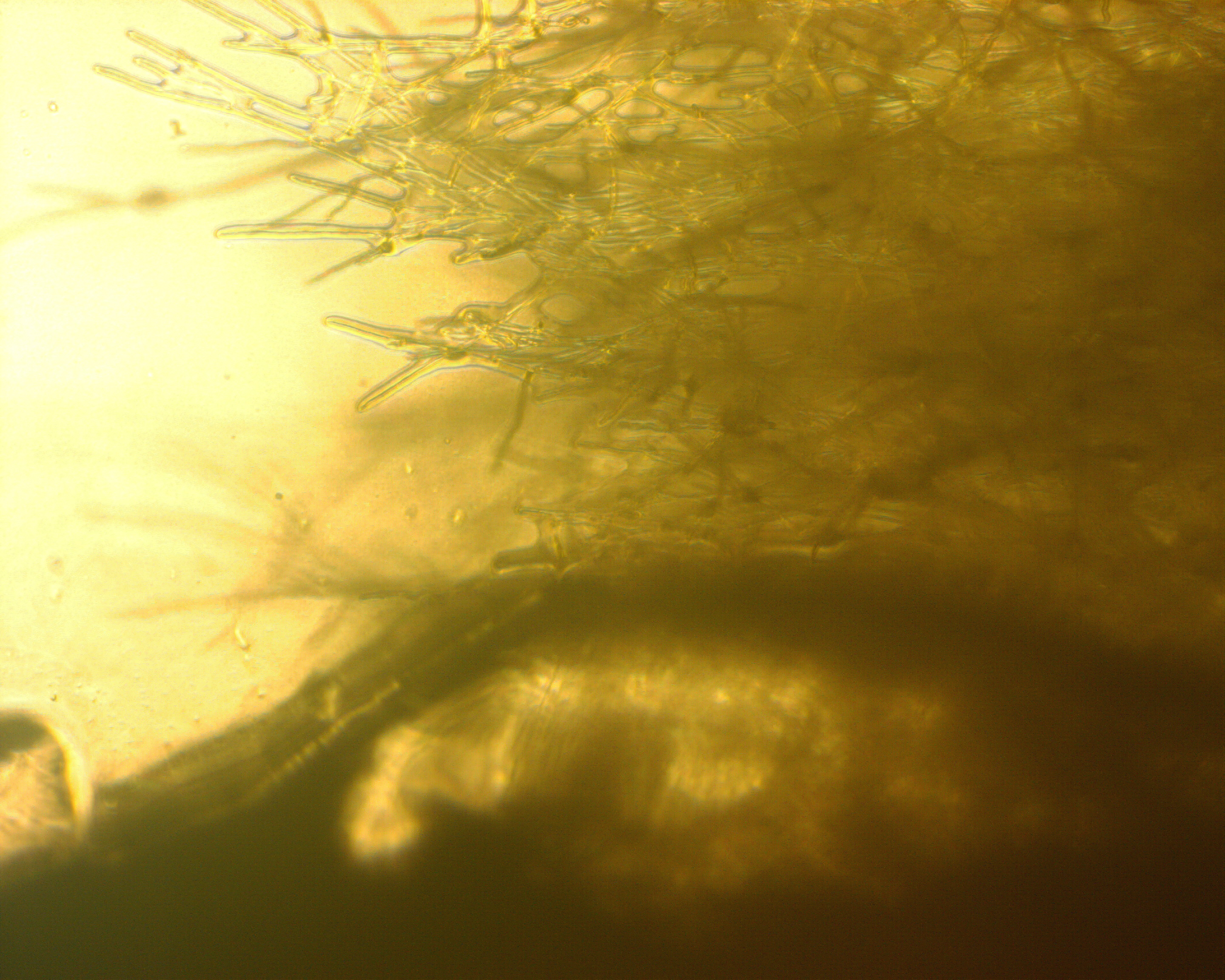

Supplement: Supplementary file 4 — Supplementary Material 4. [file 12896_2024_859_MOESM4_ESM.zip › ax invitroassay/img_shahed002.jpg]

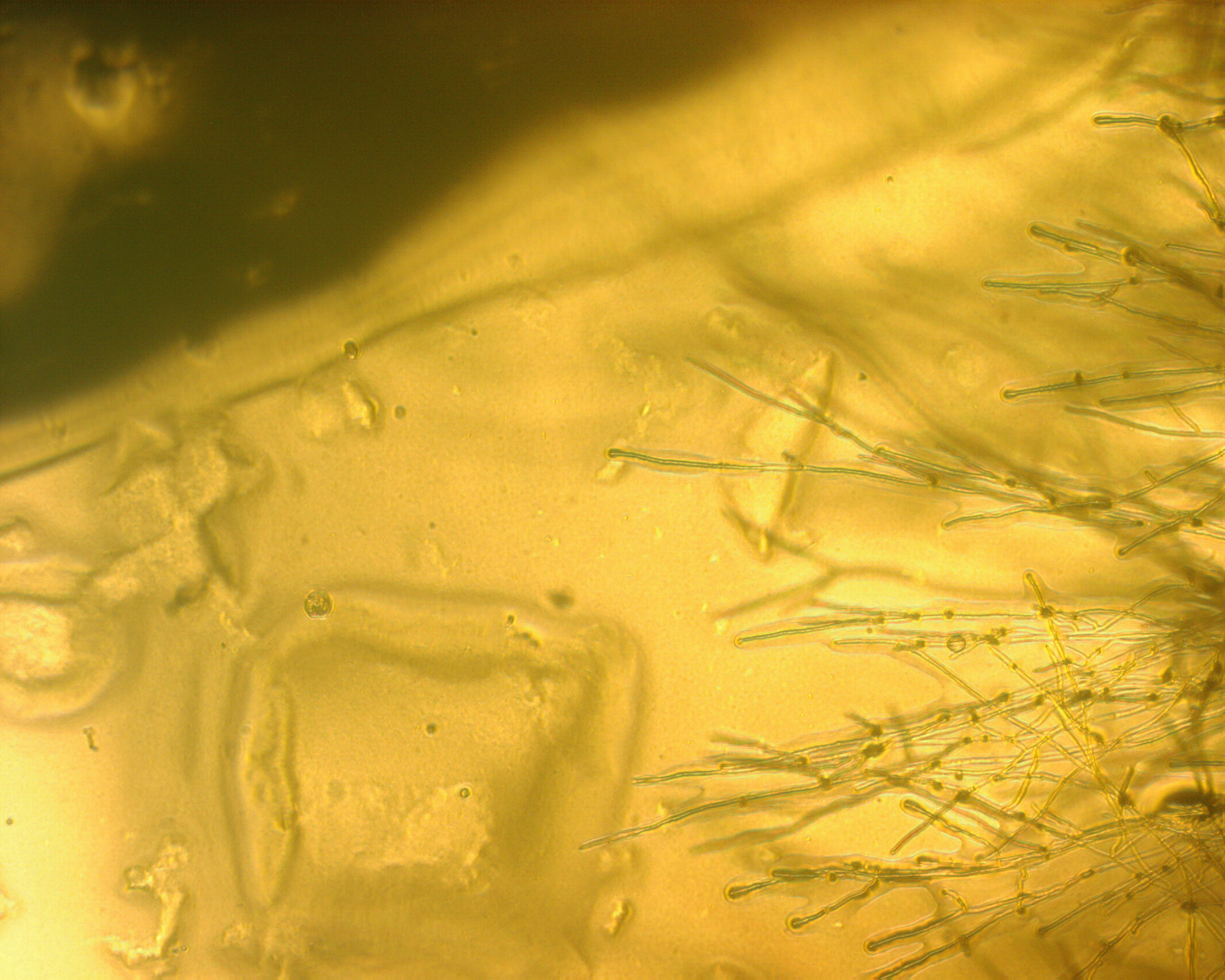

Supplement: Supplementary file 4 — Supplementary Material 4. [file 12896_2024_859_MOESM4_ESM.zip › ax invitroassay/img_tra001.jpg]

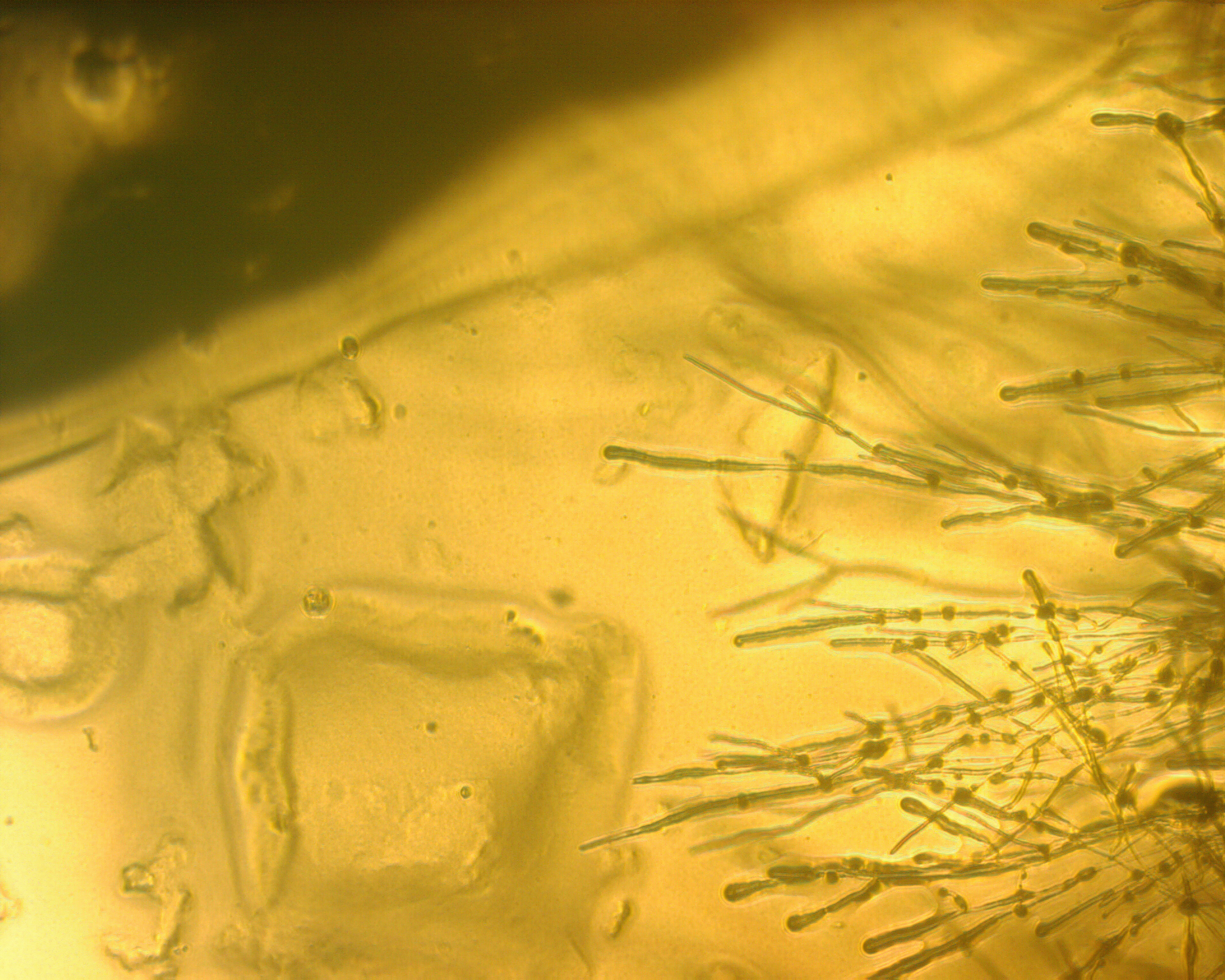

Supplement: Supplementary file 4 — Supplementary Material 4. [file 12896_2024_859_MOESM4_ESM.zip › ax invitroassay/img_tra003.jpg]

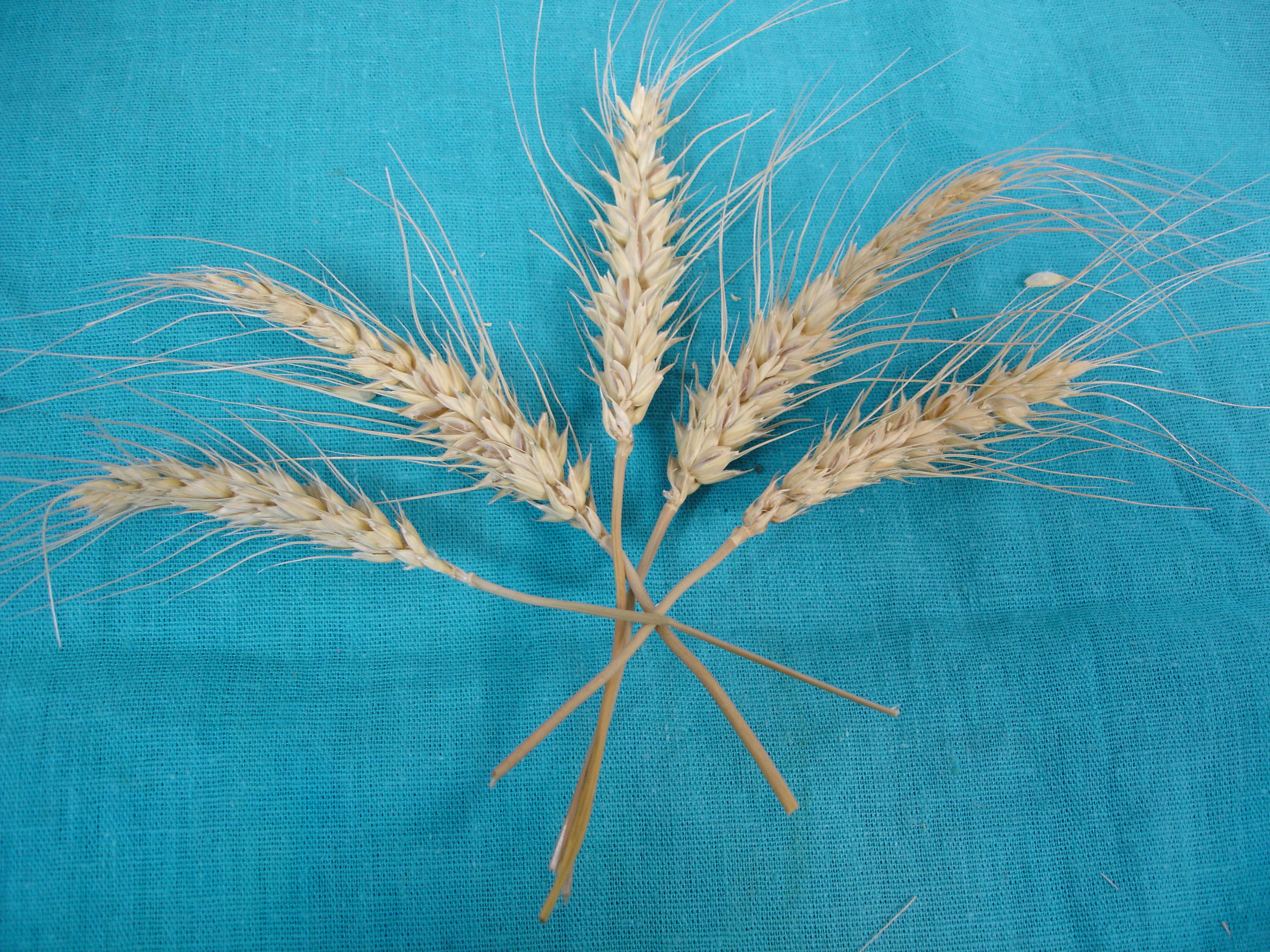

Supplement: Supplementary file 5 — Supplementary Material 5. [file 12896_2024_859_MOESM5_ESM.zip › ax گیاه/a.JPG]

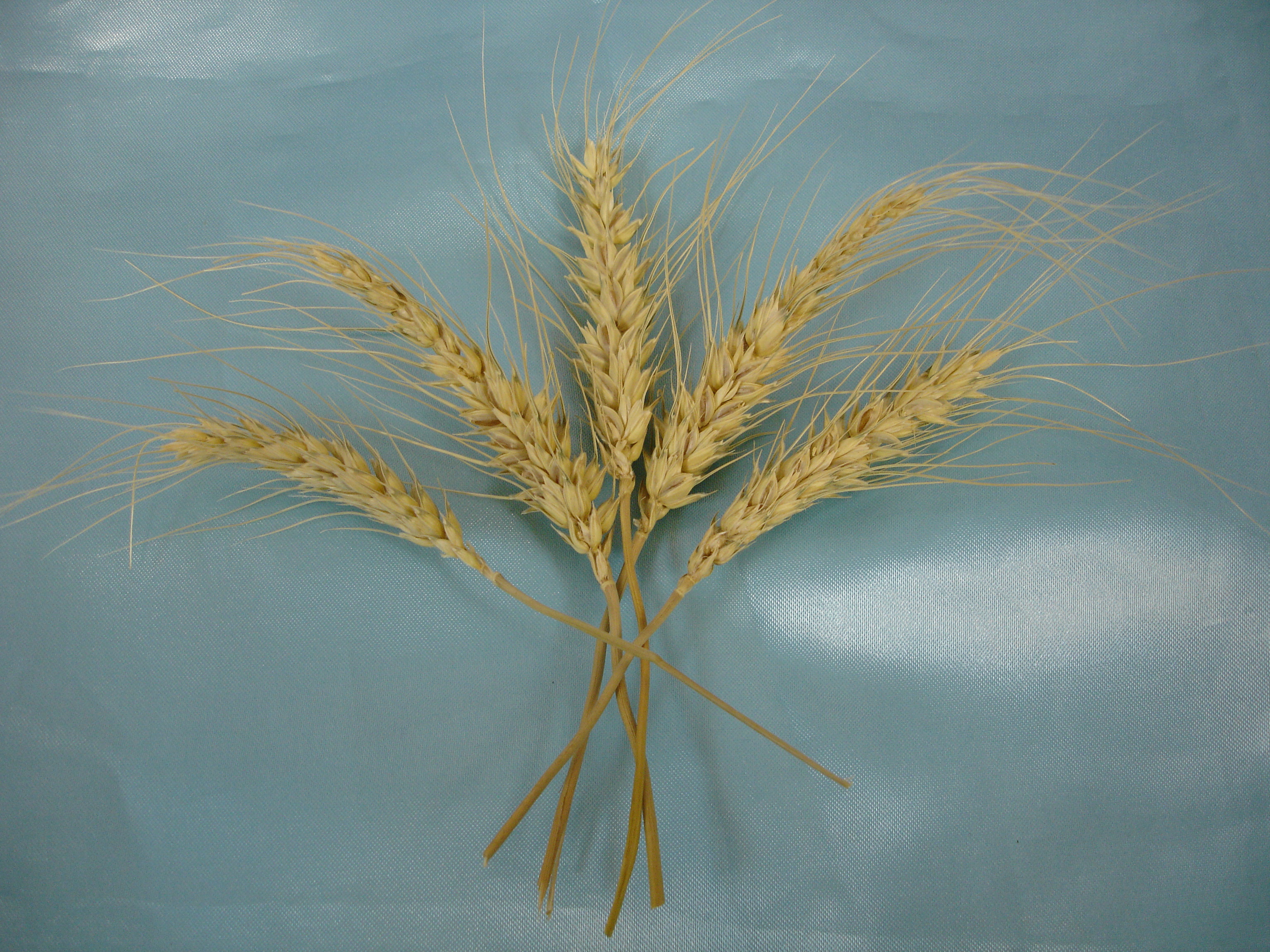

Supplement: Supplementary file 5 — Supplementary Material 5. [file 12896_2024_859_MOESM5_ESM.zip › ax گیاه/b.JPG]

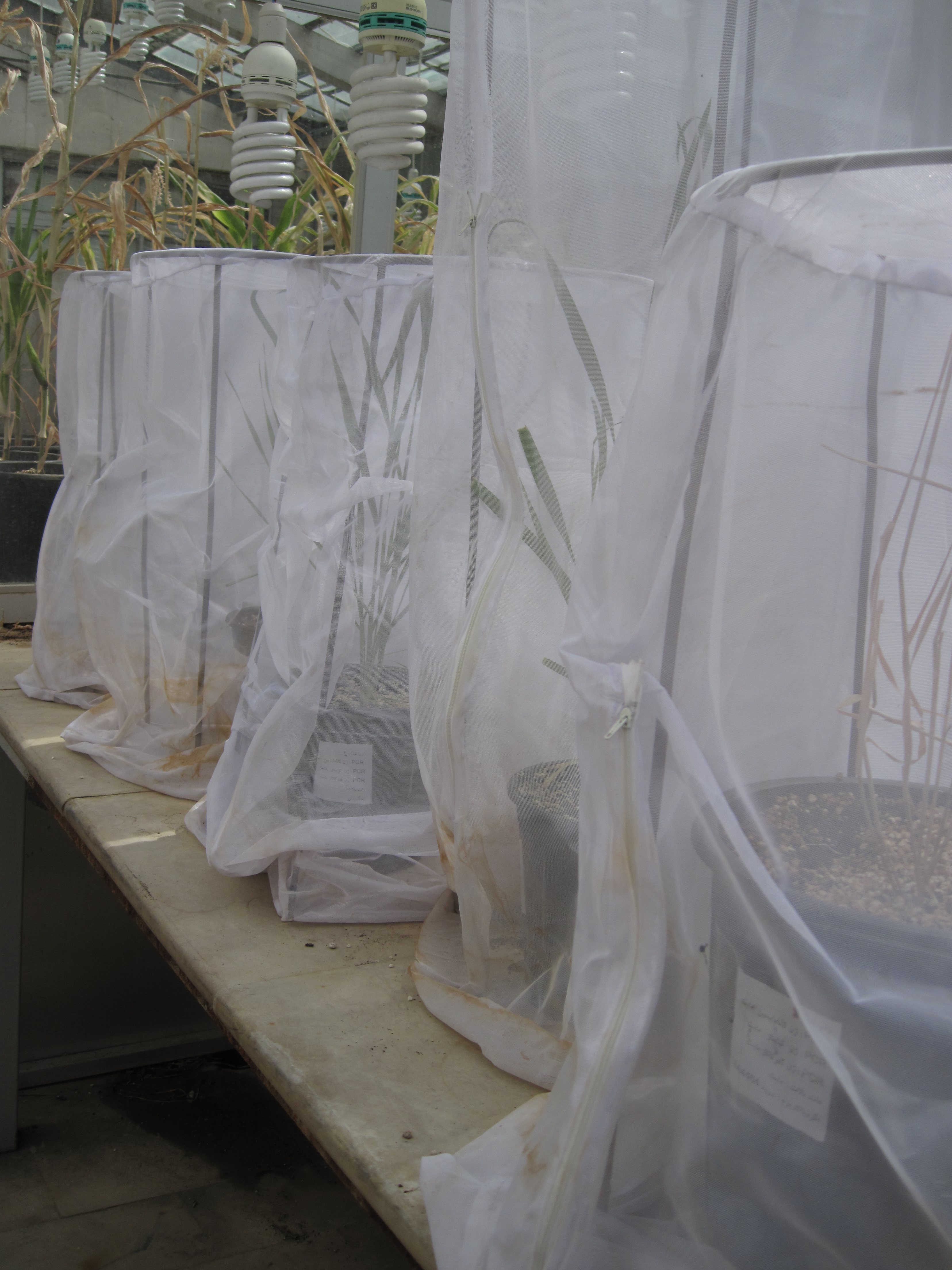

Supplement: Supplementary file 5 — Supplementary Material 5. [file 12896_2024_859_MOESM5_ESM.zip › ax گیاه/IMG_0407.JPG]

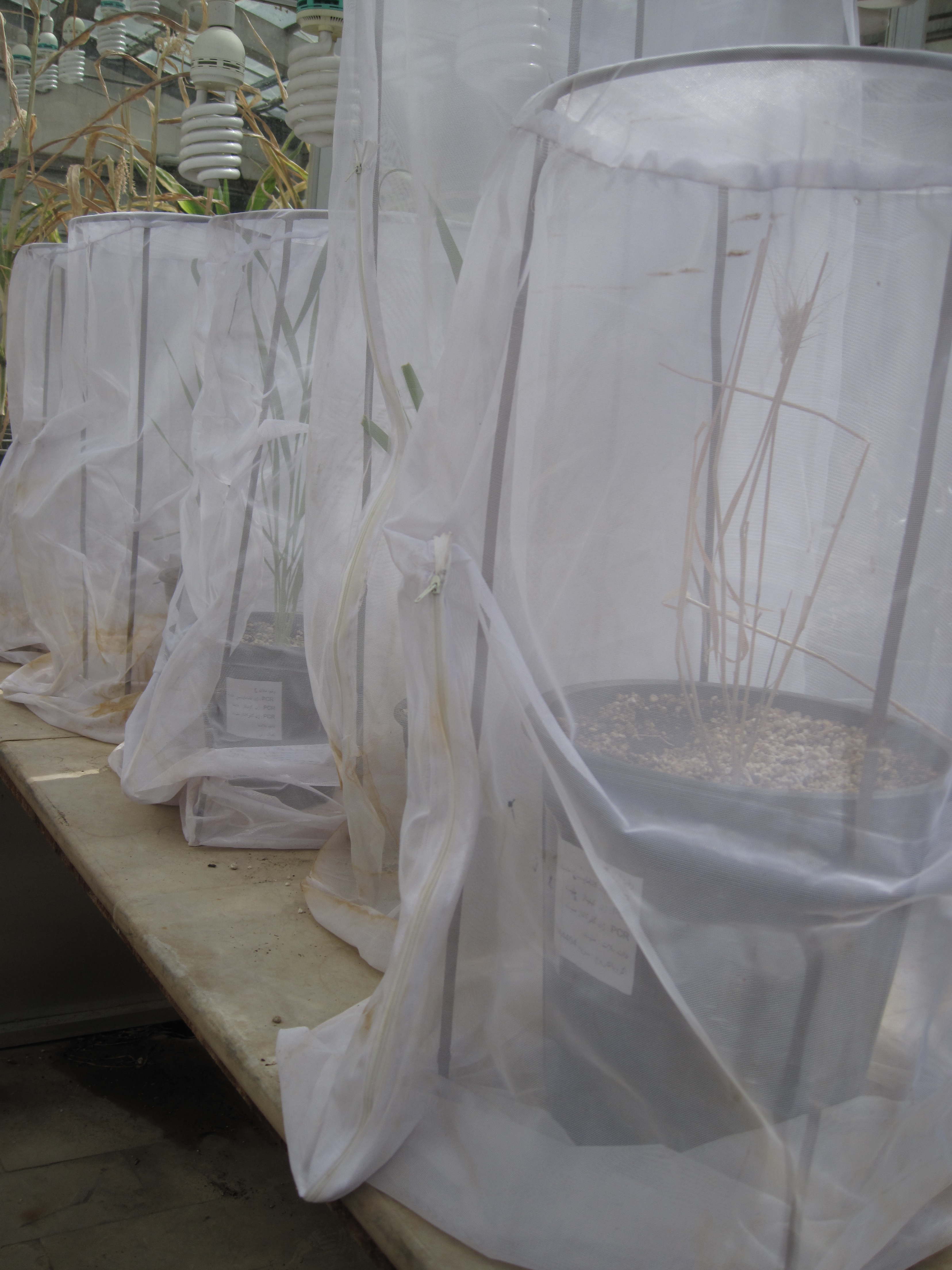

Supplement: Supplementary file 5 — Supplementary Material 5. [file 12896_2024_859_MOESM5_ESM.zip › ax گیاه/IMG_0408.JPG]

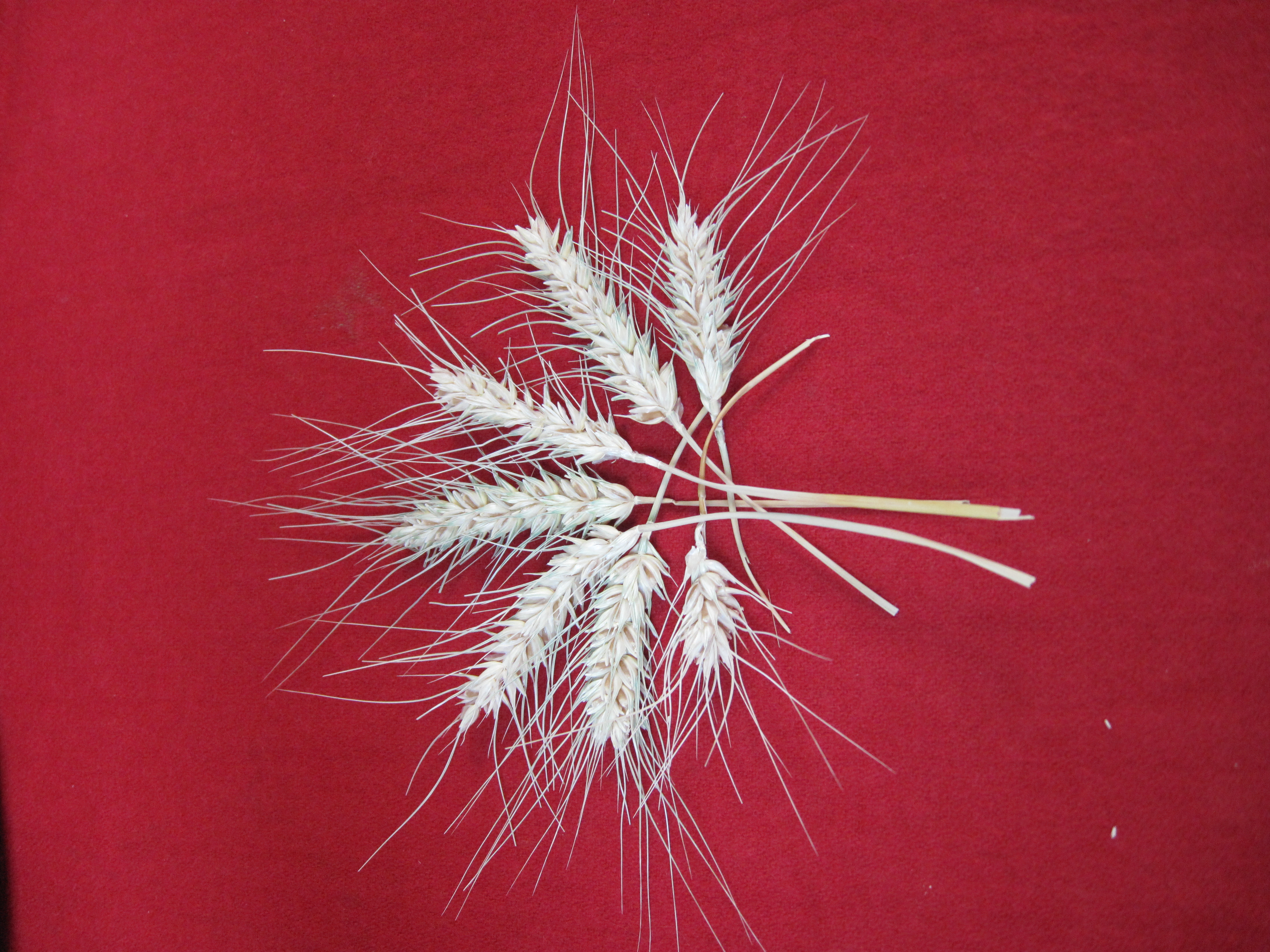

Supplement: Supplementary file 5 — Supplementary Material 5. [file 12896_2024_859_MOESM5_ESM.zip › ax گیاه/IMG_0427.JPG]
